# Health impact and cost-effectiveness of vaccination using potential next-generation influenza vaccines in Thailand: a modelling study

**DOI:** 10.1136/bmjgh-2024-015837

**Published:** 2024-11-18

**Authors:** Simon R Procter, Naomi R Waterlow, Sreejith Radhakrishnan, Edwin van Leeuwen, Aronrag Meeyai, Ben S Cooper, Sunate Chuenkitmongkol, Yot Teerawattananon, Rosalind M Eggo, Mark Jit

**Affiliations:** 1 Department of Infectious Disease Epidemiology, London School of Hygiene and Tropical Medicine, London, UK; 2 School of Biodiversity, One Health and Veterinary Medicine, University of Glasgow, Glasgow, UK; 3 UK Health Security Agency, London, UK; 4 Centre for Tropical Medicine and Global Health, University of Oxford, Nuffield Department of Medicine, Oxford, UK; 5 Mahidol-Oxford Tropical Medicine Research Unit, Mahidol University, Bangkok, Thailand; 6 National Vaccine Institute, Nonthaburi, Thailand; 7 Health Intervention and Technology Assessment Program, Ministry of Health, Muang, Thailand; 8 Saw Swee Hock School of Public Health, National University of Singapore, Singapore

**Keywords:** Vaccines, Health economics, Respiratory infections, Immunisation, Global Health

## Abstract

**Introduction:**

Thailand was one of the first low- and middle-income countries to publicly fund seasonal influenza vaccines, but the lack of predictability in the timing of epidemics and difficulty in predicting the dominant influenza subtypes present a challenge for existing vaccines. Next-generation influenza vaccines (NGIVs) are being developed with the dual aims of broadening the strain coverage and conferring longer-lasting immunity. However, there are no economic evaluations of NGIVs in Thailand.

**Methods:**

We estimated the health impact and cost-effectiveness of NGIVs in Thailand between 2005 and 2009 using a combined epidemiological and economic model. We fitted the model to data on laboratory-confirmed influenza cases and then simulated the number of influenza infections, symptomatic cases, hospitalisations and deaths under different vaccination scenarios based on WHO-preferred product characteristics for NGIVs. We used previous estimates of costs and disability adjusted life years (DALYs) for influenza health outcomes to estimate incremental net monetary benefit, vaccine threshold prices and budget impact.

**Results:**

With the current vaccine programme, there were an estimated 61 million influenza infections. Increasing coverage to 50% using improved vaccines reduced infections to between 23 and 57 million, and with universal vaccines to between 21 and 49 million, depending on the age groups targeted. Depending on the comparator, threshold prices for NGIVs ranged from US$2.80 to US$12.90 per dose for minimally improved vaccines and US$24.60 to US$69.90 for universal vaccines.

**Conclusion:**

Influenza immunisation programmes using NGIVs are anticipated to provide considerable health benefits and be cost-effective in Thailand. However, although NGIVs might even be cost-saving in the long run, there could be significant budget implications for the Thai government even if the vaccines can be procured at a substantial discount to the maximum threshold price.

**WHAT IS ALREADY KNOWN ON THIS TOPIC**

- While there have been cost-effectiveness studies on the implementation of seasonal influenza vaccines in many countries, several next-generation influenza vaccines (NGIVs) are currently in clinical trials.
- Previous studies have assessed the cost-effectiveness of NGIVs in the USA, the UK and Kenya, but there have been no studies in Thailand, which was one of the first low-/middle-income countries to introduce publicly funded influenza vaccination.

**WHAT THIS STUDY ADDS**

- This is the first cost-effectiveness study of NGIVs in Thailand and the first such study for an upper-middle-income country. It is also the first analysis to assess the impact of such vaccines in a Southeast Asia setting, where seasonal influenza epidemiology differs from countries included in previous studies.
- Our study shows that NGIVs could potentially yield a substantial reduction in influenza burden in Thailand and are likely to be cost-effective.
- However, our analysis also indicates the large up-front financial impact that adoption of more costly vaccines could incur, particularly if the introduction of NGIVs is accompanied by higher population coverage.

**HOW THIS STUDY MIGHT AFFECT RESEARCH, PRACTICE OR POLICY**

- Our study adds to the global evidence, showing that NGIVs are likely to be cost-effective and bolsters the investment case for the development of such vaccines.
- However, the upfront budget implications for self-financing countries of implementing NGIVs and expanding their coverage could pose a significant barrier to adoption. This suggests that such vaccines could need staged introduction and/or innovative pricing and financing mechanisms to be accessible in most low- and middle-income countries.
- As well as being of interest to policymakers in Thailand and other countries, our analysis will also contribute to the evidence base for the full value of vaccine assessment for improved influenza vaccines being led by the WHO.

## Introduction

 In Thailand, seasonal influenza is an important cause of morbidity and mortality, responsible for an estimated six deaths per 100 000 population each year, with most deaths occurring in people aged over 60 years.^1 2^ Influenza also causes a substantial economic burden in Thailand.^3^ Current prevention strategies include annual vaccination of healthcare workers, people aged 65 and over, children aged 6–23 months, pregnant women and high-risk individuals. However, except for healthcare workers, coverage in these groups remains low (between 2% and 20%).^4^ A recent study also suggested that seasonal vaccination of older school-age children with trivalent live-attenuated influenza vaccine could avert a substantial burden of influenza, in particular through indirect protection of the elderly and is likely to be cost-effective.^5^

In contrast to temperate countries that typically experience a single seasonal influenza epidemic in winter, in tropical countries seasonality is less clear, although in Thailand, some authors have suggested that a typical pattern is for two annual peaks, with a major peak between June and August and a minor peak between October and February.^6^ Lack of predictability in the timing of epidemics and the added difficulty in predicting the dominant influenza subtypes,^7^ present a challenge for existing vaccines, which must be reformulated each year to target specific strains. Thus, the impact of vaccination in a given season depends on the effectiveness of vaccines against the strains in circulation at the time. Furthermore, the immunity from current vaccines may be short-lived,^8^ and vaccine effectiveness may thus be sensitive to differences in the timing of seasonal epidemics and annual vaccination campaigns.

To address these challenges, next-generation influenza vaccines (NGIVs) are being developed with the dual aims of broadening the strain coverage and conferring longer-lasting immunity.^9^ Previous studies have reported the cost-effectiveness of vaccine programmes using NGIVs in the UK, Kenya and the USA.^10^,^13^ However, Thailand differs from those settings in terms of influenza seasonality, average income and healthcare provision. Additionally, Thailand was one of the first middle-income countries to introduce influenza vaccination, so it may be an early adopter of NGIVs when they are available. The price at which NGIVs could be cost-effective in Thailand is an important consideration for facilitating investment in NGIV development as well as guiding decision-makers when such vaccines become available. The WHO recommends that these economic considerations are incorporated into a full value of vaccines assessment,^14^ which should be undertaken while new vaccines are still in development.

The aim of this study is to assess the potential impact and cost-effectiveness of using NGIVs in the current vaccine programme in Thailand, as well as programmes with expanded age targeting of children, and to investigate how these are influenced by the different characteristics of potential NGIVs. This work contributes to the full value of influenza vaccine assessments conducted to inform recommendations by the WHO.

## Methods

**Figure 1:**
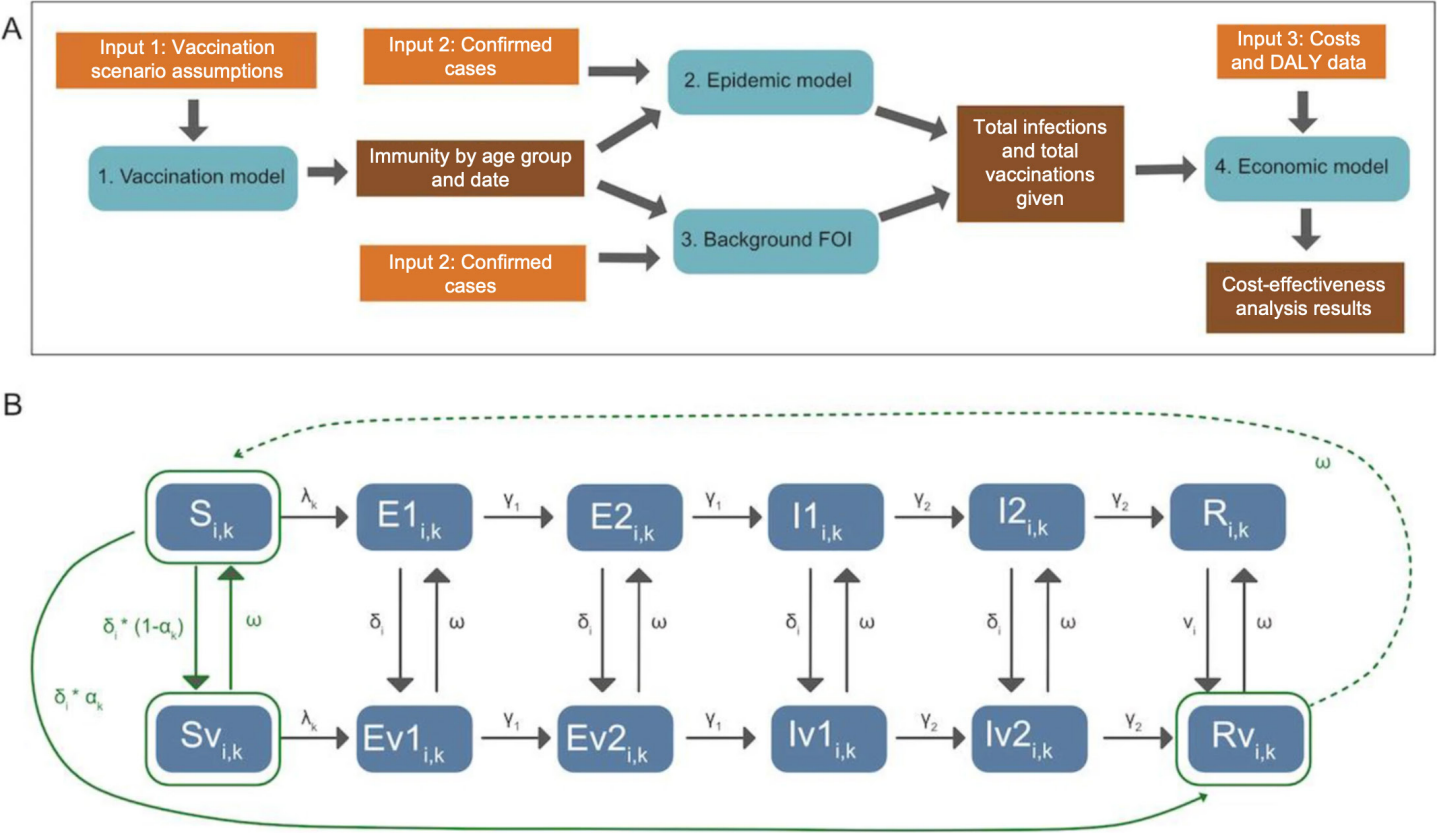
Overview of modelling framework and transmission model structure adapted from Waterlow *et al*. ^10^ (A) Flowchart showing the combined vaccination, transmission and economic model used in the analysis. Orange shows inputs to each part of the model, and brown shows the outputs from each step. (B) The transmission model used in this work is stratified by age (1) and influenza strain (k) and has two arms, representing unvaccinated and vaccinated groups. The E and I compartments have two sequential compartments to model Erlang (gamma) delay distributions. DALY, disability adjusted life year; FOI, force of infection.

We explored the health and economic impact of different seasonal influenza vaccines in Thailand using a pre-existing multi-step modelling framework^10 11^ and reported our findings in line with the updated Consolidated Health Economic Evaluation Reporting Standards checklist (online supplemental appendix table 1). The modelling framework is based on an extension of the *FluEvidenceSynthesis* package^15^ that combines a vaccination model, an epidemic model and an economic model (figure 1A and online supplemental appendix figure 1) to estimate the number of influenza infections and cost-effectiveness of influenza vaccination under different scenarios. Model input parameters for the different modelling components are summarised in online supplemental appendix table 2 and online supplemental appendix table 3.

The compartmental model structures for the vaccine model and the epidemic model are shown in figure 1. The models were stratified into six age groups: <2 years, 2–5 years, 6–11 years, 12–17 years, 18–59 years and 60+ years, and run separately for A(H1N1), A(H3N3) and B(combined) influenza subtypes. The Thai population in each age group was based on estimates reported by the United Nations World Population Prospects.^16^ Contact patterns between these age groups were accounted for using the contact matrix derived by and reported by Meeyai *et al* based on empirical contact-survey data.^5^

The vaccination model consists of Susceptible (S), Susceptible-Vaccinated (Sv) and Recovered-Vaccinated (Rv) compartments (highlighted green in figure 1). Individuals in the model are assumed to be vaccinated regardless of prior immunity status, with an effectively immunised proportion of vaccinated individuals entering Rv and the complement entering Sv, from where vaccine protection wanes exponentially by transitioning back to the S compartment at a rate dependent on the vaccine scenario.

The epidemic model has a susceptible, exposed, infected and recovered structure, which is further stratified by vaccination status. The output from the vaccination model was used to define the proportion of the population in each of the S, Sv and Rv compartments at the start of each epidemic period.

We used data from six different subtype-specific epidemic periods (online supplemental appendix figure 2) during the years 2005–2009. The epidemic periods were identified using monthly data on laboratory-confirmed influenza cases by subtype^6^ and applying prespecified criteria: (1) periods started with two consecutive months of increasing cases, (2) monthly cases during the period were above the median for the whole time series and (3) periods ended with two consecutive months of decreasing cases. For each epidemic period, we estimated the reporting rate, susceptibility, transmissibility and the initial number of infections at the start of the epidemic period by fitting these data using a binomial likelihood for the monthly number of reported cases. We used the adaptive Markov Chain Monte Carlo algorithm in the *FluEvidenceSynthesis* R package.^15^ Posterior distributions of the fitted parameters are shown in online supplemental appendix figure 3.

Since there were some influenza cases during inter-epidemic periods, we added a background force of infection for each influenza subtype, modelled using a Poisson distribution fitted to the number of cases during these periods. This ensured that cases (or vaccine impact) were included throughout the period under study. The number of infections using the fitted models for each subtype combining the epidemic and non-epidemic periods is shown in online supplemental appendix figure 4.

To estimate the number of deaths due to influenza, we used previously estimated age-, subtype- and season-specific influenza per capita death rates,^1^ and combined these with the predicted number of symptomatic cases without vaccination from our fitted model to estimate the probability of death among symptomatic cases (online supplemental appendix figure 5). The proportion of influenza cases that were symptomatic was modelled using beta distributions parameterised using data for each virus subtype from Carrat *et al*.^17^

Similarly, to calculate the proportion of symptomatic cases that were treated as inpatients (IPs) and outpatients (OPs), we used age-specific data on the annual number of reported OP and IP cases of influenza-like illnessfrom Chittaganpitch *et al*^6^ divided by the baseline predicted number of symptomatic cases and modelled this as beta distributions.

We modelled health outcomes under different vaccine effectiveness (VE) assumptions and different coverage assumptions (table 1). For some vaccine scenarios, the effectiveness of the vaccine was assumed to depend on whether or not the vaccine was matched to the circulating virus subtype in a given season. Whether or not vaccines were considered matched or mismatched for a given year was based on a comparison of influenza vaccine strains against surveillance data on the circulating strains in Thailand.^6^ For current seasonal vaccines, we assumed 70% VE in matched seasons and 40% VE in mismatched seasons. Since NGIVs are still in development and their efficacy is unknown, we explored different assumptions about potential NGIVs guided by the WHO-preferred product characteristics.^14^

**Table 1:** Assumptions about different next-generation influenza vaccine scenarios developed from the WHO preferred product characteristics^27^ and alternative coverage scenarios

Vaccine scenario	Mismatched seasons?	Vaccine effectiveness (matched/mismatched)	Immunity duration
Current vaccines	Yes	70%/40%	6 months
Improved vaccines (minimal)	Yes	70%/40%	1 year
Improved vaccines (efficacy)	Yes	90%/70%	2 years
Improved vaccines (breadth)	No	70%/70%	3 years
Universal vaccines	No	90%/90%	5 years

Coverage scenario	Vaccine coverage by age-group					
	<2 years	2–5 years	6–11 years	12–17 years	18–60 years	60+ years
S1: Current policy (existing coverage)	2.84%*	0%	0%	%	5.15%†	30%
S2: Current policy (50% coverage)	37.5%*	0%	0%	0%	7.05%†	50%
S3: Current policy+children 2–5 years (50% coverage)	37.5%	50%	0%	0%	7.05%	50%
S4: Current policy+children 2–17 years (50% coverage)	37.5%	50%	50%	50%	7.05%	50%

* Adjusted by a factor of 0.75 to reflect that the current policy targets children aged 6–24 months, but the model age band is 0–24 months, so, for example, 37.5% coverage from 0–24 months corresponds to 50% coverage for 6–24 months.

† Reflects coverage among eligible risk groups, so, for example, 7.05% coverage of the entire 18-year to 60-year population reflects a 50% coverage among eligible adult risk groups.

For each of the vaccine scenarios, we modelled the impact under four coverage scenarios chosen based on discussion with one of the authors (YT), who is a leader of a national health technology assessment agency in Thailand (table 1). These scenarios were: S1: current vaccination policy with existing age-targeting and coverage^18^; S2: current vaccination policy with improved coverage of 50% in all target groups and expansion of the age-targeting beyond the current vaccination policy to include children aged 2–5 years (scenario S3) or 2–17 years (scenario S4).

For the cost-effectiveness analysis, we used costs and disability adjusted life year (DALY) inputs from Meeyai *et al*.^5^ We adopted a partial societal perspective, including direct medical and non-medical costs and indirect time costs, but did not include any productivity loss associated with deaths due to influenza. Direct medical costs of treatment for influenza-related illness were estimated for non-medically attended cases based on the cost of over-the-counter medication, for OP care based on the unit cost of attendance, and for IP care based on unit costs and length-of-stay. Direct vaccination costs comprised delivery costs, including logistics and vaccine administration, as well as the cost of purchasing the vaccine. Direct non-medical costs included expenses incurred by patients and their relatives for travel to the healthcare facilities for vaccination and/or treatment for influenza. Indirect costs encompassed the opportunity costs based on average income associated with time spent seeking care. All costs were inflated to the year 2022 using the Thailand GDP deflator^19^ and were converted to US dollars using the average annual exchange rate.^20^ Future costs were discounted at an annual rate of 3%. Cost inputs are summarised in online supplemental appendix table 3).

For non-fatal health outcomes, we included years lived with disability (YLDs) for symptomatic cases, cases requiring OP treatment and IP cases (online supplemental appendix table 3). To fully capture the impact of influenza deaths, we used a lifetime time horizon and estimated average discounted years of life lost (YLLs) for deaths in each age group using the model using UN life tables^16^ and assuming that within each age group, the risk of influenza mortality was proportional to age-specific all-cause mortality. Future DALYs were also discounted at 3%, but 0% discounting of DALYs was explored in sensitivity analysis in line with current guidelines.^21^

We calculated the incremental costs, DALYs averted and net monetary benefit (NMB) compared with existing coverage of current vaccines, assuming that all vaccines had the same price of US$3.20 (102 Baht) per dose, which is similar to the price of the current trivalent seasonal vaccine in Thailand (860 000 doses procured at a reported cost of 88 million Baht).^22^ Since the market price of future vaccines is currently unknown, we also calculated the threshold price at which NGIVs would be cost-effective based on a cost-effectiveness threshold of US$5003 (160 000 Baht) per DALY averted, which is the most recently updated recommended value in Thailand.^23^ We calculated threshold prices using two different comparators: (1) the threshold price compared with *existing* coverage of the current vaccination policy (ie, scenario S1) using current vaccines and (2) the threshold price of NGIVs compared with current vaccines under the *same* coverage scenario. Thus, using a comparator (1) captures the effect of *both* the improved characteristics of NGIVs *and* expanded age-targeting and/or coverage, whereas using a comparator (2) captures the benefit of the improved characteristics of the NGIV, having already accounted for the impact that would be achieved with higher coverage/or broader age-targeting of current vaccines. To capture the combined uncertainty in model inputs, for each analysis, we ran 1000 simulations, sampling from each parameter distribution and used this to calculate the 95% uncertainty range (UR).

To assess the potential budget impact of different NGIVs and coverage scenarios, we estimated the net cost of the vaccine programme to the healthcare system each year after accounting for cost-savings from averted influenza cases. In this analysis, we assumed that NGIVs would be purchased at a discount to the estimated threshold vaccine price (compared with the existing coverage of the current vaccine programme) and based the level of discount on the ratio of the current seasonal vaccine price (US$3.20) to the estimated threshold price for current vaccines.

## Results

We fitted our model to the number of monthly reported influenza cases during the years 2005 to 2009 (online supplemental appendix figure 2). Without vaccination, we estimated there would be 74 million (95% UR: 67–80 million) influenza infections in Thailand between April 2005 and March 2009 (online supplemental appendix table 4). Seasonal vaccination of children with the existing (2022) coverage (scenario S1) using current vaccines was predicted to reduce this to 61 million (55–67 million), while improving coverage of the current programme to 50% (scenario S2) further reduced this to 55 million (49–60 million) infections. Vaccination targeting all children aged 0–17 years (scenario S4) achieved the highest impact but also required the most doses. With the use of improved vaccines, cumulative infections fell to between 24 million (22–25 million) and 57 million (50–62 million), and with universal vaccines to between 21 (20–22 million) and 49 million (43–53 million) infections, depending on the scenario. The annual number of vaccine doses under different vaccine and coverage scenarios is shown in online supplemental appendix figure 6, and the corresponding cumulative impact on influenza infections over time is shown in online supplemental appendix figure 7.

**Figure 2:**
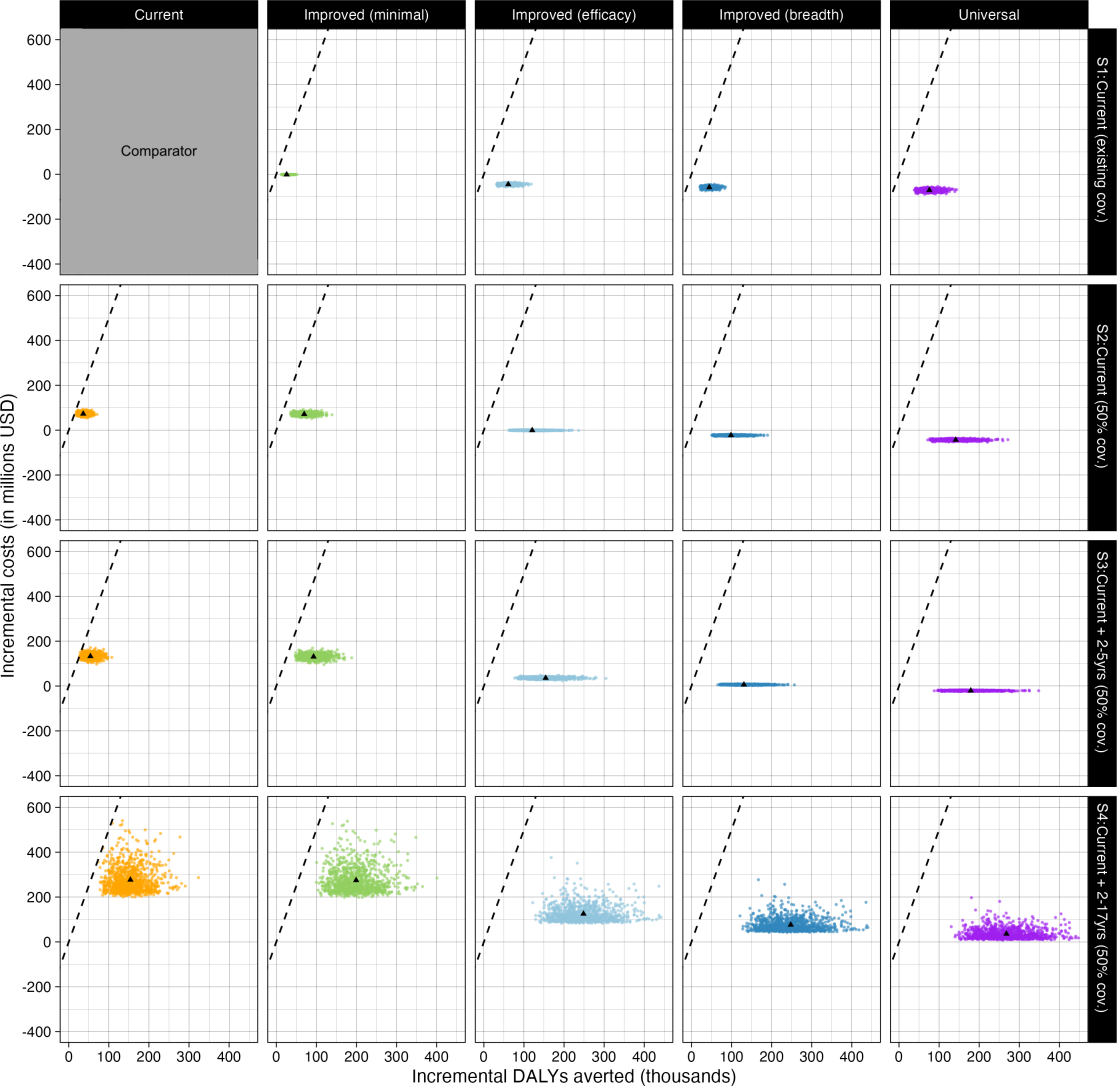
Cost-effectiveness plane showing incremental costs and incremental DALYs averted for different next-generation influenza vaccine types under different coverage scenarios compared to the existing coverage of current vaccines. Dashed lines show the guideline cost-effectiveness threshold of 160 000 Baht per DALY averted. DALY, disability adjusted life year.

Replacing current vaccines with NGIVs while maintaining existing coverage was cost-saving under the assumption of fixed vaccine prices (figure 2). However, expansion of vaccine coverage could either increase costs or be cost-saving depending on the coverage and vaccine characteristics. Improved (efficacy), improved (breadth) and universal vaccines were more likely to be cost-saving or to have lower incremental costs due to the reduction in the number of doses needed within a given coverage scenario as well as a reduction in influenza-related healthcare costs. Compared with current seasonal vaccination, the number of DALYs averted was greater for all NGIV types, and for a given coverage scenario, it increased in the order: improved (minimal) vaccines, improved (breadth) vaccines, improved (efficacy) vaccines and were highest for universal vaccines. For expanded coverage scenarios, the number of DALYs averted by NGIVs was smaller when the comparator was the same coverage of current vaccines (comparator (2), (online supplemental appendix figure 8).

**Figure 3:**
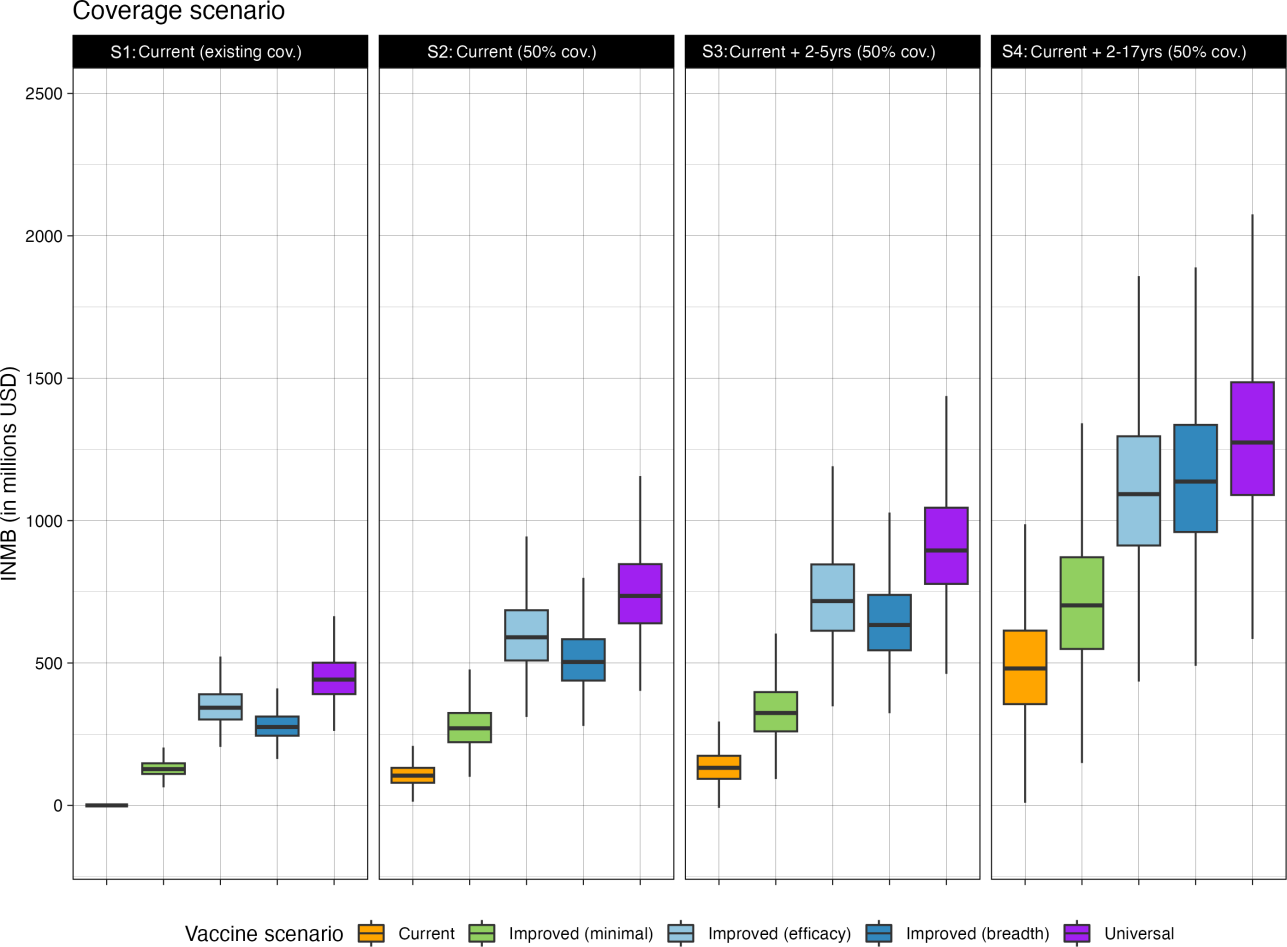
Incremental net monetary benefit of different next-generation influenza vaccine types and coverage scenarios compared to existing coverage of current vaccines.

Overall, at the current price of US$3.20 per dose, we found expanding paediatric vaccination with current seasonal vaccines was cost-effective for all age groups, consistent with the previous findings.^5^ Using the same price assumption, the different NGIV scenarios were all cost-effective (figure 3). For a given type of NGIV, the NMB varied with coverage scenario, with higher NMB achieved in scenarios with higher coverage and broader age-targeting. Overall, the largest NMB is achieved when including children aged 2–17 years (scenario S4, figure 3), indicating that at current vaccine prices, it would be cost-effective to extend vaccine coverage to the widest age group. For expanded coverage scenarios, the NMB was smaller for NGIVs when the comparator was the same coverage of current vaccines (comparator (2), online supplemental appendix figure 9), but the NMB was larger when DALYs were discounted by 0% (online supplemental appendix figure 10).

In reality, it is unlikely that new vaccines will become available at the same cost as existing vaccines; however, NGIVs could remain cost-effective at higher prices. Depending on the coverage scenario, the threshold price below which the vaccines remained cost-effective compared with existing coverage using current vaccines (comparator (1) in table 2) varied from US$8.68 to US$12.90 per dose for minimally improved vaccines to US$49.30 to US$69.90 per dose for universal vaccines. When NGIVs were compared against the equivalent coverage of current vaccines (comparator (2) in table 2), then the threshold prices were lower, ranging from US$2.80 to US$8.68 for minimally improved vaccines to between US$24.60 and US$69.00 per dose for universal vaccines. Discounting DALYs at 0% increased threshold vaccine prices (online supplemental appendix table 5).

**Table 2:** Threshold prices in US dollars below which different types of vaccines and coverage scenarios become cost-effective using the recommended cost-effectiveness threshold in Thailand of 160 000 per disability adjusted life year averted

Comparator	Vaccine scenario	Coverage scenario			
		S1: current policy (existing coverage)	S2: current policy (50% coverage)	S3: current policy+2–5 year olds (50% coverage)	S4: Current policy+2–17 year olds (50% coverage)
(1) Current vaccines and current coverage (scenario S1)	Current	Comparator (1)	5.41 (2.56, 9.29)	5.98 (2.59, 10.7)	10.1 (4.89, 17.5)
	Improved (minimal)	8.68 (5.61, 13.5)	12.2 (7.14, 20.0)	12.0 (6.54, 20.1)	12.9 (6.85, 21.5)
	Improved (efficacy)	35.3 (24.0, 53.0)	37.7 (24.3, 57.3)	34.9 (22.1, 53.2)	28.3 (17.8, 44.7)
	Improved (breadth)	34.1 (23.4, 50.0)	39.9 (26.1, 60.3)	38.8 (24.7, 59.6)	36.0 (23.0, 56.3)
	Universal	69.0 (47.3, 101)	69.9 (46.4, 104)	64.2 (42.1, 96.3)	49.3 (32.0, 75.4)
(2) Current vaccines with the same coverage scenario (ie, comparing within the same column)	Current	Comparator (2)	Comparator (2)	Comparator (2)	Comparator (2)
	Improved (minimal)	8.68 (5.61, 13.5)	6.82 (4.37, 10.6)	6.01 (3.84, 9.22)	2.80 (1.77, 4.28)
	Improved (efficacy)	35.3 (24.0, 53.0)	29.2 (20.1, 42.5)	25.5 (17.5, 36.6)	12.4 (8.50, 17.6)
	Improved (breadth)	34.1 (23.4, 50.0)	29.3 (20.5, 42.1)	27.1 (19.1, 38.5)	16.3 (11.3, 23.0)
	Universal	69.0 (47.3, 101)	56.7 (39.5, 81.6)	49.8 (35.1, 70.8)	24.6 (17.4, 34.7)

Threshold prices are presented based on two comparators: (1) the threshold price compared with existing coverage of the current vaccination policy (ie, scenario S1) using current vaccines and (2) the threshold price of next-generation influenza vaccines compared with current vaccines under the same coverage scenario.

**Figure 4:**
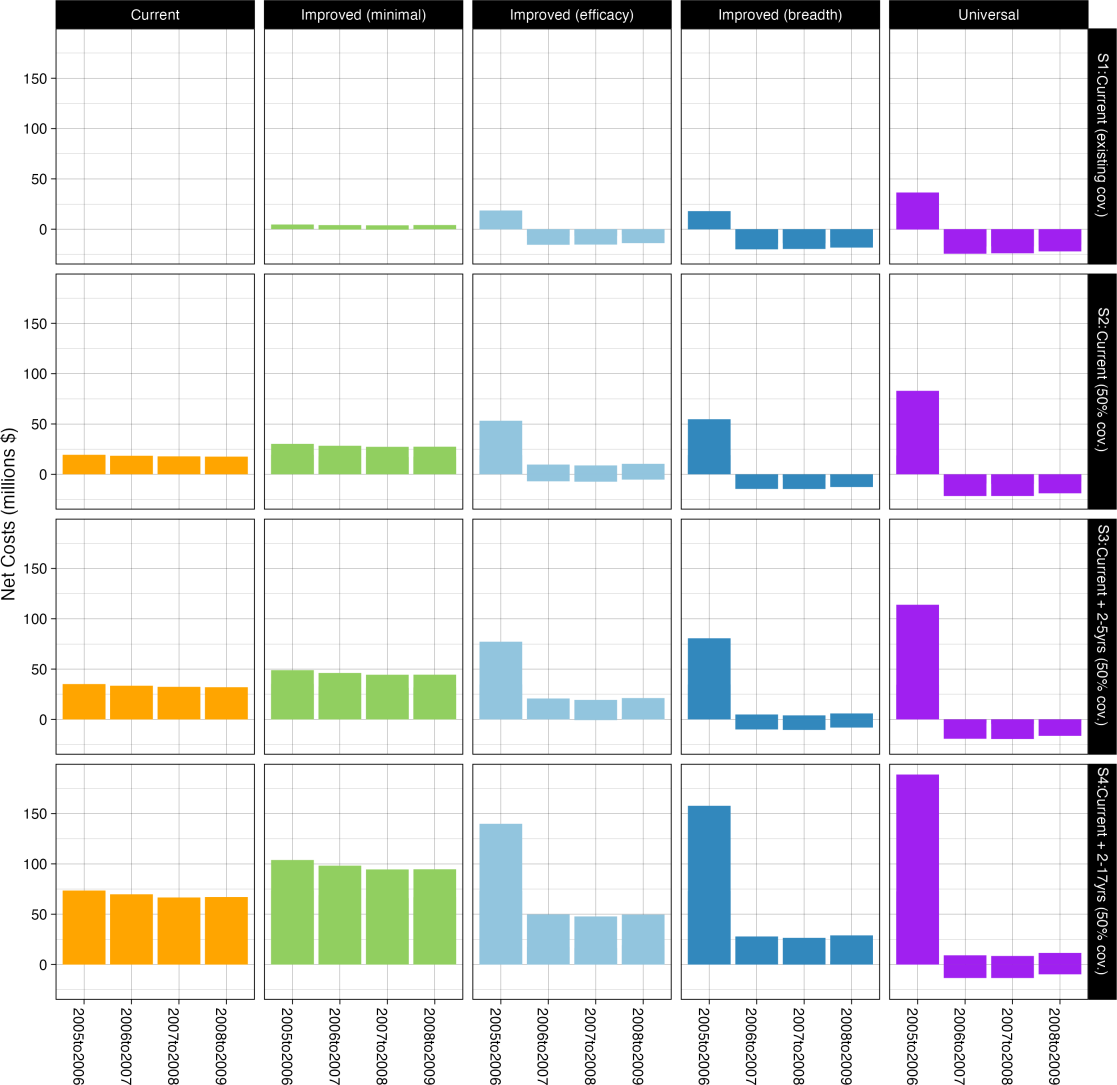
Annual budget impact of different vaccine types and different age targeting strategies under the assumption that different vaccines are priced at a similar discount relative to the estimated threshold price as current vaccines.

Although NGIVs may be cost-effective at higher prices, it is also important to consider the potential budget impact of implementing a large vaccine programme. The annual net costs under different vaccine scenarios, assuming that vaccines can be purchased at an ~80% discount to the estimated threshold price (compared with existing coverage of current vaccines), are shown in figure 4. As expected, the budget impact is larger for scenarios that have higher coverage and target larger numbers of children. For current seasonal and improved (minimal) vaccines, the annual cost remains broadly constant, reflecting that the population is assumed to require annual vaccination (online supplemental appendix figure 6). In contrast, for improved efficacy, improved breadth and universal vaccines, the number of doses is reduced after the first year due to the longer duration of vaccine-acquired immunity. As a consequence, these vaccines have a large upfront cost in year one due to their higher price, which then drops substantially in subsequent years and may even become cost-saving compared with the current vaccines.

## Discussion

Similar to our previous studies in Kenya^10^ and the UK,^11^ we found that NGIVs could be highly cost-effective and more cost-effective than current inactivated trivalent vaccines being used in Thailand, with universal vaccines being the most cost-effective. Indeed, we found that greatly expanding the target age range of the current policy by giving vaccines to children aged up to 17 years would be cost-effective. Adding children aged 2–17 years to the current vaccine policy had the highest net monetary benefit of the scenarios that we explored. Furthermore, vaccinating a wide age range (eg, 0–17 years) is needed to prevent the majority of influenza cases across all age groups in Thailand (assuming coverage of around 50%). Our results are consistent with a previous cost-effectiveness evaluation of Trivalent Influenza Vaccine (TIV) in Thailand,^5^ which found that paediatric TIV is cost-effective at an acquisition cost of 11.3 international dollars per dose, with the most cost-effective target group being aged 12–17 years.

However, our cost-effectiveness results for NGIVs assume the same price per dose as current vaccines, which is unlikely given the additional investment that manufacturers need to make to develop them and the likely market advantage they will have over current vaccines. Although higher uptake of NGIVs due to their enhanced characteristics might potentially yield economies of scale in vaccine production, thereby lowering their costs in the long run. In a threshold analysis, we found that, compared with the existing policy using current vaccines, NGIVs priced between US$12.90 (for improved (minimal) vaccines) and US$49.30 (for universal vaccines) could remain cost-effective with a vaccine policy expanded to include children aged 2–17 years. This is well above the current price for seasonal influenza vaccines in Thailand (US$3.20) and reflects the excellent cost-effectiveness profile of the current influenza vaccination programme in Thailand. At the same time, our analyses highlight the enhanced health benefit that NGIVs could have over current vaccines and the savings that would be obtained from not having to deliver vaccines so often due to greater duration of protection.

Despite the favourable cost-effectiveness, the short-term budget impact of NGIVs could be substantial. In the highest budget impact scenario, increasing coverage to 50% and expanding age-targeting to 2–17 year olds using universal influenza vaccines costing US$13.06 a dose could cost over US$150 million in the year of vaccine introduction, which is about 3.3% of Thailand’s current healthcare budget.^24^ Although cost-saving in later years could mean that the opportunity cost of vaccine introduction in the year of introduction (in terms of other healthcare displaced) could be substantially higher than the current cost-effectiveness threshold suggests. Indeed, such high upfront financial costs would likely be too great for the Thai government to bear. However, there may be ways to reduce the initial high outlay, such as staggering new vaccine introduction and expansion of eligible populations over several years and negotiating price reductions for bulk purchases from suppliers.

Our conclusions are subject to several limitations. Like most other economic evaluations of influenza vaccines, the study may underestimate the cost-effectiveness of vaccines, as we did not incorporate many broader socioeconomic benefits of influenza vaccination, such as reduction in productivity losses, antimicrobial resistance and non-respiratory health outcomes like cardiac conditions that have been linked to influenza.^25 26^ Given the policy emphasis and current capacity of the Thai immunisation system, we only considered scenarios involving mass paediatric vaccination. In particular, we did not consider the possibility of vaccinating high-risk groups (such as people of any age with clinical conditions putting them at higher risk of influenza complications) or healthcare workers.

Also, we based our assumptions about NGIV efficacy on the WHO-preferred product characteristics, which are relatively simple, likely due to the lack of detailed experimental data on these vaccines at present. In particular, influenza vaccines often afford different levels of protection against viral transmission, mild disease, severe disease and death, but we have assumed all-or-nothing sterilising immunity, that is, successfully immunised individuals are totally protected against infection until antibodies wane, whereas individuals suffering from primary vaccine failure receive no protection. If vaccine protection against viral transmission is lower than protection against disease, this would lower the cost-effectiveness of paediatric vaccine programmes for which a substantial part of the impact is through indirect protection of the vulnerable.

Lastly, we used data from 2005 to 2009 in order to be able to capture prevaccination influenza epidemiology. Hence the results do not reflect changes to influenza epidemiology that may have occurred following the 2009 influenza pandemic and the more recent COVID-19 pandemic, for example, such as the disappearance of the influenza B/Yamagata lineage.

## Conclusions

Influenza immunisation programmes using NGIVs are anticipated to provide considerable health benefits and be cost-effective in Thailand. However, although NGIVs might even be cost-saving in the long run, there could be significant budget implications for the Thai government even if the vaccines can be procured at a substantial discount to the maximum threshold price.

## Supplementary material

10.1136/bmjgh-2024-015837 online supplemental appendix 1

## Data Availability

Data are available in a public, open access repository.
