# Supplementary material for: Health impact and cost-effectiveness of vaccination using potential next-generation influenza vaccines in Thailand: a modelling study
Source: BMJ Glob Health. 2024 Nov 18;9(11):e015837. doi: 10.1136/bmjgh-2024-015837 (PMC11574519; doi:10.1136/bmjgh-2024-015837)
Supplement: online supplemental appendix 1 [file bmjgh-9-11-s001.pdf]

Supplementary Appendix to Health impact and cost-effectiveness of vaccination using potential next-generation influenza vaccines in Thailand: a modelling study.

Simon R Procter[1]*, Naomi R Waterlow[1]*, Sreejith Radhakrishnan[1,2], Edwin van Leeuwen[1,3], Aronrag Meeyai[4], Ben S Cooper[4,5], Sunate Chuenkitmongkol[6], Yot Teerawattananon[7,8], Rosalind M Eggo[1†], Mark Jit[1†]

*contributed equally
†contributed equally

Correspondence: simon.procter@lshtm.ac.uk

1. Department of Infectious Disease Epidemiology, London School of Hygiene and Tropical Medicine, London WC14 7HT, United Kingdom
2. School of Biodiversity, One Health and Veterinary Medicine, University of Glasgow, Glasgow G61 1QH, United Kingdom
3. Modelling and Economics Unit and NIHR Health Protection Research Unit, UK Health Security Agency, London NW9 5EQ, United Kingdom
4. Centre for Tropical Medicine and Global Health, Nuffield Department of Medicine, University of Oxford, Oxford, United Kingdom
5. Mahidol-Oxford Tropical Medicine Research Unit, Faculty of Tropical Medicine, Mahidol University, Bangkok, Thailand
6. National Vaccine Institute, Nonthaburi, Thailand
7.Health Intervention and Technology Assessment Program, Ministry of Public Health, Nonthaburi, Thailand
8. Saw Swee Hock School of Public Health, National University of Singapore, Singapore

A. CHEERS checklist 3

Supplementary Table 1: Updated Consolidated Health Economic Evaluation Reporting Standards (CHEERS) checklist. 3

B. Supplementary methods 6

B.1 Transmission model 6

B.1.1 Vaccine Model Equations 6

B.1.2 Epidemic Model Equations 6

B.1.3 Transmission model input parameters 7

B.2 Health impact and economic model 8

B.2.1 Model structure 8

B.2.2 Health impact and economic model parameters 9

B.3 Epidemic model fitting 12

B.3.1 Model fitting and priors 12

B.3.2 Model data 12

B.3.3 Posterior parameter distributions 14

B.3.4 Simulations combining epidemic and non-epidemic periods. 15

B.4 Risk of death given symptomatic infection 16

C. Supplementary results 17

C.1 Vaccine doses given under different scenarios 17

C.2 Impact of different vaccine and coverage scenarios 18

C.3 Cost-Effectiveness 22

C.4 Net Monetary Benefit 23

C.5 Threshold Vaccine Prices 25

A. CHEERS checklist

Supplementary Table 1: Updated Consolidated Health Economic Evaluation Reporting Standards (CHEERS) checklist.

Topic	No.	Item	Location where item is reported
**Title and abstract**			
Title	1	Identify the study as an economic evaluation and specify the interventions being compared.	See title on page 1
Abstract	2	Provide a structured summary that highlights context, key methods, results, and alternative analyses.	See abstract on page 2
**Introduction**			
Background and objectives	3	Give the context for the study, the study question, and its practical relevance for decision making in policy or practice.	See introduction on pages 4 and 5
**Methods**			
Health economic analysis plan	4	Indicate whether a health economic analysis plan was developed and where available.	Not applicable as not RCT based
Study population	5	Describe characteristics of the study population (such as age range, demographics, socioeconomic, or clinical characteristics).	Methods page 5
Setting and location	6	Provide relevant contextual information that may influence findings.	Methods pages 5 to 9
Comparators	7	Describe the interventions or strategies being compared and why chosen.	Methods page 9
Perspective	8	State the perspective(s) adopted by the study and why chosen.	Methods page 8
Time horizon	9	State the time horizon for the study and why appropriate.	Methods page 8
Discount rate	10	Report the discount rate(s) and reason chosen.	Methods page 8
Selection of outcomes	11	Describe what outcomes were used as the measure(s) of benefit(s) and harm(s).	Methods pages 8 and 9
Measurement of outcomes	12	Describe how outcomes used to capture benefit(s) and harm(s) were measured.	Supplementary table 3 / previously described in Meeyai et al.
Valuation of outcomes	13	Describe the population and methods used to measure and value outcomes.	Supplementary table 3 / previously described in Meeyai et al.

Measurement and valuation of resources and costs	14	Describe how costs were valued.	Supplementary table 3 / previously described in Meeyai et al.
Currency, price date, and conversion	15	Report the dates of the estimated resource quantities and unit costs, plus the currency and year of conversion.	Methods page 8 / supplementary table 3
Rationale and description of model	16	If modelling is used, describe in detail and why used. Report if the model is publicly available and where it can be accessed.	Methods page 9 (include link to open access Github repository)
Analytics and assumptions	17	Describe any methods for analysing or statistically transforming data, any extrapolation methods, and approaches for validating any model used.	Methods and Supplementary Appendix B
Characterising heterogeneity	18	Describe any methods used for estimating how the results of the study vary for subgroups.	Not applicable
Characterising distributional effects	19	Describe how impacts are distributed across different individuals or adjustments made to reflect priority populations.	Not applicable
Characterising uncertainty	20	Describe methods to characterise any sources of uncertainty in the analysis.	Methods para 10 on page 8 and para 17 on page 10, and S1 Appendix A2.1 and A2.2
Approach to engagement with patients and others affected by the study	21	Describe any approaches to engage patients or service recipients, the general public, communities, or stakeholders (such as clinicians or payers) in the design of the study.	Authorship includes Thai policymakers who provided input to the study
**Results**			
Study parameters	22	Report all analytic inputs (such as values, ranges, references) including uncertainty or distributional assumptions.	Table 1, Supplementary Tables 2 and 3 and Github repository
Summary of main results	23	Report the mean values for the main categories of costs and outcomes of interest and summarise them in the most appropriate overall measure.	Results pages 9 and 10
Effect of uncertainty	24	Describe how uncertainty about analytic judgments, inputs, or projections affect findings. Report the effect of choice of discount rate and time horizon, if applicable.	Uncertainty distributions are shown in figure 2, uncertainty intervals are reported in main text, figure 3, table 2. Scenario analyses are reported in supplementary results.
Effect of engagement with patients and	25	Report on any difference patient/service recipient, general public, community, or	Not applicable, no patients involved in the study

others affected by the study		stakeholder involvement made to the approach or findings of the study	
**Discussion**			
Study findings, limitations, generalisability, and current knowledge	26	Report key findings, limitations, ethical or equity considerations not captured, and how these could affect patients, policy, or practice.	Discussion on pages 14 to 16
**Other relevant information**			
Source of funding	27	Describe how the study was funded and any role of the funder in the identification, design, conduct, and reporting of the analysis	Acknowledgements on page 17
Conflicts of interest	28	Report authors conflicts of interest according to journal or International Committee of Medical Journal Editors requirements.	Declaration of interests on page 17

B. Supplementary methods

B.1 Transmission model

The compartmental model structure for the vaccine and epidemiological transmission models are shown in Figure 1 of the main text.

B.1.1 Vaccine Model Equations

$$\frac{dS_i}{dt} = -\delta_i * S_i + \omega * (Sv_i + Rv_i)$$
$$\frac{dSv_i}{dt} = \delta_i * S_i * (1 - \alpha_i) - \omega * Sv_i$$
$$\frac{dRv_i}{dt} = \delta_i * S_i * \alpha_i - \omega * Rv_i$$

Where:

i - age group;

S - Susceptible;

Sv - Vaccinated and susceptible;

Sv_i - Vaccinated and immune;

v - vaccination rate;

α - vaccine efficacy;

$1/\omega$ - duration of vaccine immunity

B.1.2 Epidemic Model Equations

$$\frac{dS_i}{dt} = -\lambda_i * S_i - v_i * S_i + \omega * (Sv_i + Rv_i)$$
$$\frac{dE1_i}{dt} = \lambda_i * S_i - \gamma_1 * E1_i - \delta_i * E1_i + \omega * Ev1_i$$
$$\frac{dE2_i}{dt} = \gamma_1 * E1_i - \gamma_1 * E2_i - \delta_i * E2_i + \omega * Ev2_i$$
$$\frac{dI1_i}{dt} = \gamma_1 * E2_i - \gamma_2 * I1_i - \delta_i * I1 + \omega * Iv1_i$$
$$\frac{dI2_i}{dt} = \gamma_2 * I1_i - \gamma_2 * I2_i - \delta_i * I2 + \omega * Iv2_i$$
$$\frac{dR_i}{dt} = \gamma_2 * I2_i - \delta_i * R_i + \omega * Rv2_i$$

$$\frac{dSv_i}{dt} = -\lambda_i * Sv_i + (1-\alpha_i) * \delta_i * S_i - \omega * Rv_i$$

$$\frac{dEv1_i}{dt} = \lambda_i * Sv_i - \gamma_1 * Ev1_i + \delta_i * E1_i - \omega * Ev1_i$$

$$\frac{dEv2_i}{dt} = \gamma_1 * Ev1_i - \gamma_1 * Ev2_i + \delta_i * E2_i - \omega * Ev2_i$$

$$\frac{dIv1_i}{dt} = \gamma_1 * Ev2_i - \gamma_2 * Iv1_i + \delta_i * I1 - \omega * Iv1_i$$

$$\frac{dIv2_i}{dt} = \gamma_2 * Iv1_i - \gamma_2 * Iv2_i + \delta_i * I2 - \omega * Iv2_i$$

$$\frac{dRv_i}{dt} = \gamma_2 * Iv2_i + \delta_i * R_i + \alpha_i * \delta_{i} * S_i - \omega * Rv2_i$$

Where the force of infection is given by:

$$\lambda_i = \beta * \zeta_i * \sum_{j=1}^{j=N} c_{ij} * (I1_j + I2_j + Iv1_j + Iv2_j)$$

B.1.3 Transmission model input parameters

Supplementary Table 2: Transmission model input parameters

Parameter	Symbol	Source	Value (if fixed)
Age-specific vaccination rate	δ_i	Assumption based on vaccine scenario (see table 1 of main paper)	-
Vaccine efficacy	α	Assumption based on vaccine scenario (see table 1 of main paper)	-
Age specific force of infection	λ_i	Posterior	
Transmission rate	β	Posterior	-
Contact rates between age groups i and j	c_{ij}	Meeyai et al.	-
Latency period	$2 * 1/\gamma_1$	Fixed *fluEvidenceSynthesis* package	0.8 days
Infectious Period	$2 * 1/\gamma_2$	Fixed *fluEvidenceSynthesis* package	1.8 days

Parameter	Symbol	Source	Value (if fixed)
Vaccine immunity duration	ω	Assumption based on vaccine scenario (see table 1 of main paper)	-
Age specific proportion in vaccinated compartments at start of epidemic	ηv_i	Modelled based on vaccine assumptions	-
Age specific proportion in Rv vs Sv compartments at start of epidemic	ηRv_i	Modelled based on vaccine assumptions	-
Age-specific susceptibility	ζ_i	Posterior	-

B.2 Health impact and economic model

B.2.1 Model structure

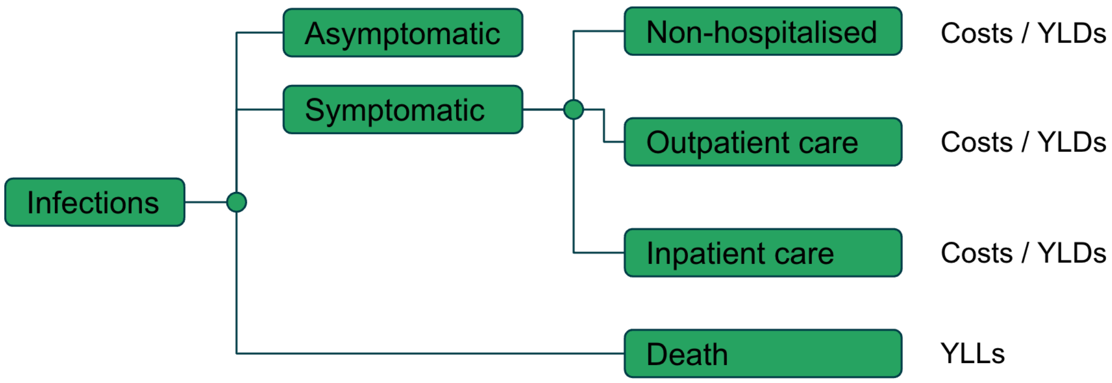

Supplementary Figure 1: Decision tree model used to estimate the total costs and total DALYs based on the projected number of infections from the vaccine and epidemiological models.

B.2.2 Health impact and economic model parameters

Supplementary Table 3: Input parameters for the health impact and economic model

Parameter	Source	Value or distribution
**Costs (all costs are presented in 2012 Thai Baht)**		
Thai GDP deflator (2022 vs 2012)	World Bank	1.122302
Exchange rate (Thai Baht per USD)	World Bank	31.98
Vaccine price per dose	https://www.nhso.go.th/news/3990	102.3 Baht
Vaccine logistics costs per dose	Meeyai et al.	Gamma(mean=18.52,SD=3.61)
Vaccine administration (ages 0 to 5 and 18+ years)	Meeyai et al.	Gamma(mean=117.63,SD=17.59)
Vaccine administration (ages 6 to 17 years)	Meeyai et al.	Gamma(mean=671.14,SD=671.14)
Cost per symptomatic case (age 0 to 1 years)	Meeyai et al.	Gamma(mean=20.19,SD=1.4)
Cost per symptomatic case (age 2 to 5 years)		Gamma(mean=14.34,SD=1.2)
Cost per symptomatic case (age 6 to 11 years)		Gamma(mean=27.09,SD=2.91)
Cost per symptomatic case (age 12 to 60+ years)		Gamma(mean=4.15,SD=0.4)
Medical cost of outpatient visit (all ages)	Meeyai et al.	Gamma(mean=371.43,SD=76.78)
Non-medical cost of outpatient visit (age 0 to 1 years)	Meeyai et al.	Gamma(mean=136.34,SD=6.1)
Non-medical cost of outpatient visit (age 2 to 60+ years)		Gamma(mean=136.15,SD=5.99)
Time cost of outpatient visit (age 0 to 1 years)	Meeyai et al.	Gamma(mean=2442.86,SD=1868.1)
Time cost of outpatient visit (age 2 to 5 years)		Gamma(mean=2400,SD=1851.64)
Time cost of outpatient visit (age 6 to 11 years)		Gamma(mean=2342.86,SD=1829.46)
Time cost of outpatient visit (age 12 to 17 years)		Gamma(mean=2457.14,SD=1873.55)
Time cost of outpatient visit (age 18 to 60 years)		Gamma(mean=1711.11,SD=975)

Parameter	Source	Value or distribution
Time cost of outpatient visit (age 60+ years)		Gamma(mean=1684.21,SD=941.5)
Medical cost of inpatient stay (age 0 to 1 years)	Meeyai et al.	Gamma(mean=7416.67,SD=1243.04)
Medical cost of inpatient stay (age 2 to 5 years)		Gamma(mean=7061.22,SD=1200.44)
Medical cost of inpatient stay (age 6 to 11 years)		Gamma(mean=6472.73,SD=1084.83)
Medical cost of inpatient stay (age 12 to 17 years)		Gamma(mean=4844.44,SD=164.05)
Medical cost of inpatient stay (age 18 to 60 years)		Gamma(mean=13483.33,SD=474.05)
Medical cost of inpatient stay (age 60+ years)		Gamma(mean=8881.58,SD=1081.03)
Non-medical cost of inpatient stay (age 0 to 1 years)	Meeyai et al.	Gamma(mean=1078.57,SD=87.77)
Non-medical cost of inpatient stay (age 2 to 5 years)		Gamma(mean=1119.23,SD=65.61)
Non-medical cost of inpatient stay (age 6 to 11 years)		Gamma(mean=975.86,SD=58.01)
Non-medical cost of inpatient stay (age 12 to 17 years)		Gamma(mean=908.89,SD=44.94)
Non-medical cost of inpatient stay (age 18 to 60 years)		Gamma(mean=880.39,SD=41.55)
Non-medical cost of inpatient stay (age 60+ years)		Gamma(mean=1100,SD=63.83)
Time cost of inpatient stay (age 0 to 1 years)	Meeyai et al.	Gamma(mean=4880,SD=220.91)
Time cost of inpatient stay (age 2 to 5 years)		Gamma(mean=4792.11,SD=112.3)
Time cost of inpatient stay (age 6 to 11 years)		Gamma(mean=4447.37,SD=108.18)
Time cost of inpatient stay (age 12 to 17 years)		Gamma(mean=4275.45,SD=43.69)
Time cost of inpatient stay (age 18 to 60 years)		Gamma(mean=4147.12,SD=25.74)
Time cost of inpatient stay (age 60+ years)		Gamma(mean=4820,SD=109.77)
DALYs for non-fatal outcomes		
DALYs per symptomatic case	Meeyai et al.	Gamma(mean=0.005,SD=0.0018)

Parameter	Source	Value or distribution
DALYs per outpatient case		Gamma(mean=0.0079,SD=0.008)
DALYs per inpatient case		Gamma(mean=0.0022,SD=0.0022)
Risk of health outcomes		
Proportion of infections that are symptomatic (AH1N1)	Carrat et al.	Beta(mean=0.702,SD=0.042)
Proportion of infections that are symptomatic (AH3N2)		Beta(mean=0.644,SD=0.012)
Proportion of infections that are symptomatic (B)		Beta(mean=0.571,SD=0.058)
Probability of death following symptomatic infection	Calculated / Meeyai et al.	Age/strain specific (see supplementary figure 2)
Probability of inpatient care if symptomatic (age 0 to 1 years)	Calculated / Meeyai et al. / Chittaganpitch et al.	Beta(mean=0.0003,SD=0.00218)
Probability of inpatient care if symptomatic (age 2 to 5 years)		Beta(mean=0.00034,SD=0.00182)
Probability of inpatient care if symptomatic (age 6 to 11 years)		Beta(mean=0.00025,SD=0.00177)
Probability of inpatient care if symptomatic (age 12 to 17 years)		Beta(mean=0.00016,SD=0.00037)
Probability of inpatient care if symptomatic (age 18 to 60 years)		Beta(mean=0.00009,SD=0.00012)
Probability of inpatient care if symptomatic (age 60+ years)		Beta(mean=0.00012,SD=0.00016)
Probability of outpatient care if symptomatic (age 0 to 1 years)	Calculated / Meeyai et al. / Chittaganpitch et al.	Beta(mean=0.00091,SD=0.00473)
Probability of outpatient care if symptomatic (age 2 to 5 years)		Beta(mean=0.00121,SD=0.0039)
Probability of outpatient care if symptomatic (age 6 to 11 years)		Beta(mean=0.00087,SD=0.00399)
Probability of outpatient care if symptomatic (age 12 to 17 years)		Beta(mean=0.00048,SD=0.00295)
Probability of outpatient care if symptomatic (age 18 to 60 years)		Beta(mean=0.00058,SD=0.00308)
Probability of outpatient care if symptomatic (age >60 years)		Beta(mean=0.00048,SD=0.00112)

B.3 Epidemic model fitting

B.3.1 Model fitting and priors

For each epidemic period we estimated the reporting rate, susceptibility, transmissibility and the initial number of infections at the start of the epidemic period by fitting to these data using a binomial likelihood for the monthly number of reported cases (see next section). We used the adaptive Markov Chain Monte Carlo (MCMC) algorithm in the FluEvidenceSynthesis R package. We ran two Markov chains using 500,000 burn-in steps, followed by 1 million steps. Posterior samples were thinned by a factor of 100 (i.e. 10,000 samples were retained.) To improve convergence of the model results we included priors on the model parameters as follows:

(i) a prior on the transmissibility, implemented as restriction on the basic reproduction number R0 which was assumed to follow a gamma distribution: Gamma(alpha=11.1, beta=2.5), which has 95% of its density between R0 = 1.6 and R0 = 3;

(ii) a restriction that the initial number of infections cannot exceed 0.01% of the population;

(iii) a prior on the susceptibility of Beta(alpha=50.2, beta=32.6) based on posterior susceptibility from Meeyai et al.

B.3.2 Model data

Epidemic periods (highlighted in grey in Supplementary Figure 3) were identified using monthly data on laboratory confirmed influenza cases by subtype and applying pre-specified criteria: (i) periods started with two consecutive months of increasing cases; (ii) monthly cases during the period were above the median for the whole time-series; (iii) periods ended with two consecutive months of decreasing cases.

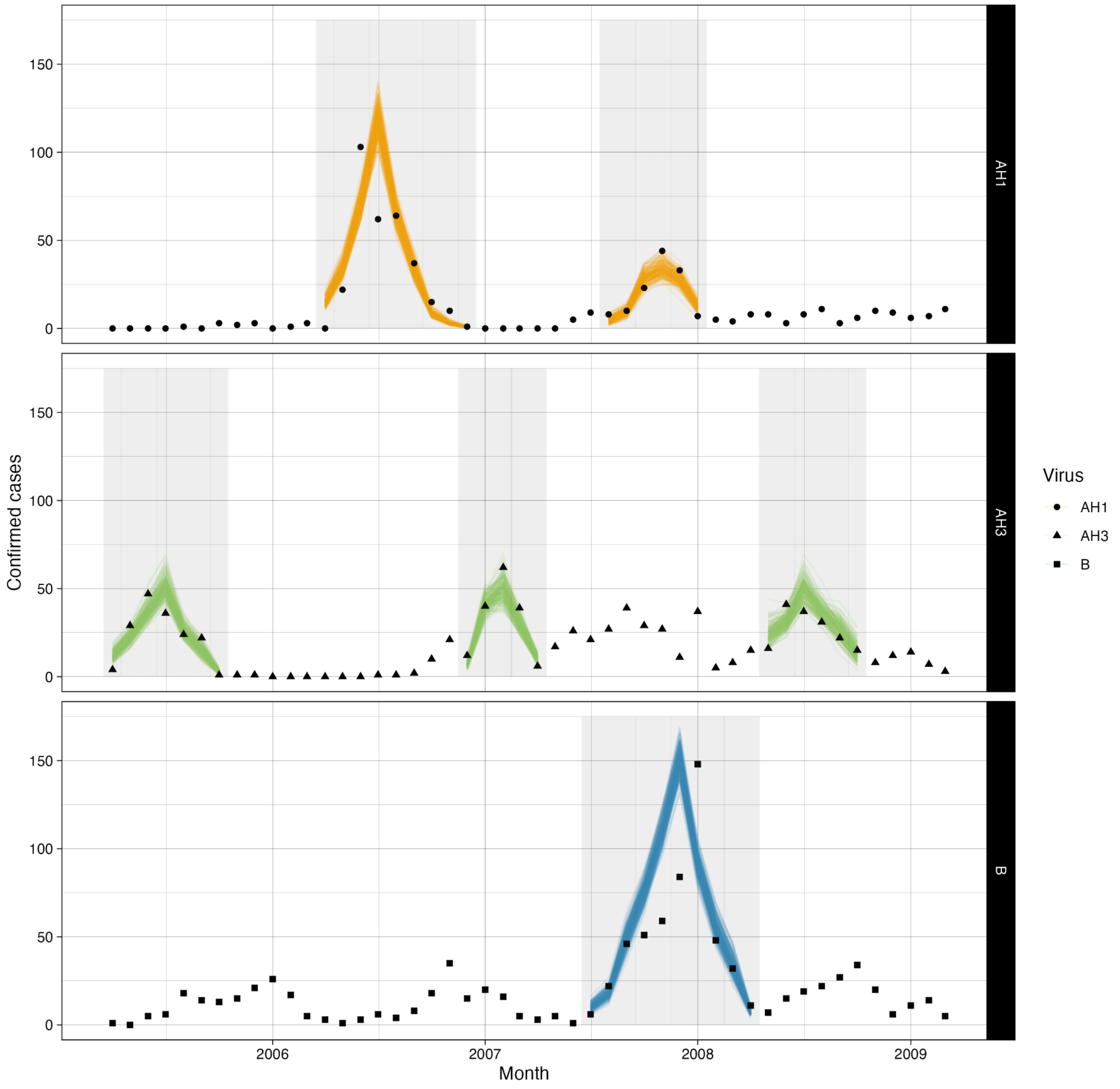

Supplementary Figure 2: Reported influenza cases. Black points show monthly laboratory confirmed influenza cases by subtype; shaded regions show months identified as being part of an epidemic included in the fitting; coloured lines show 200 simulations using posterior samples for each epidemic.

B.3.3 Posterior parameter distributions

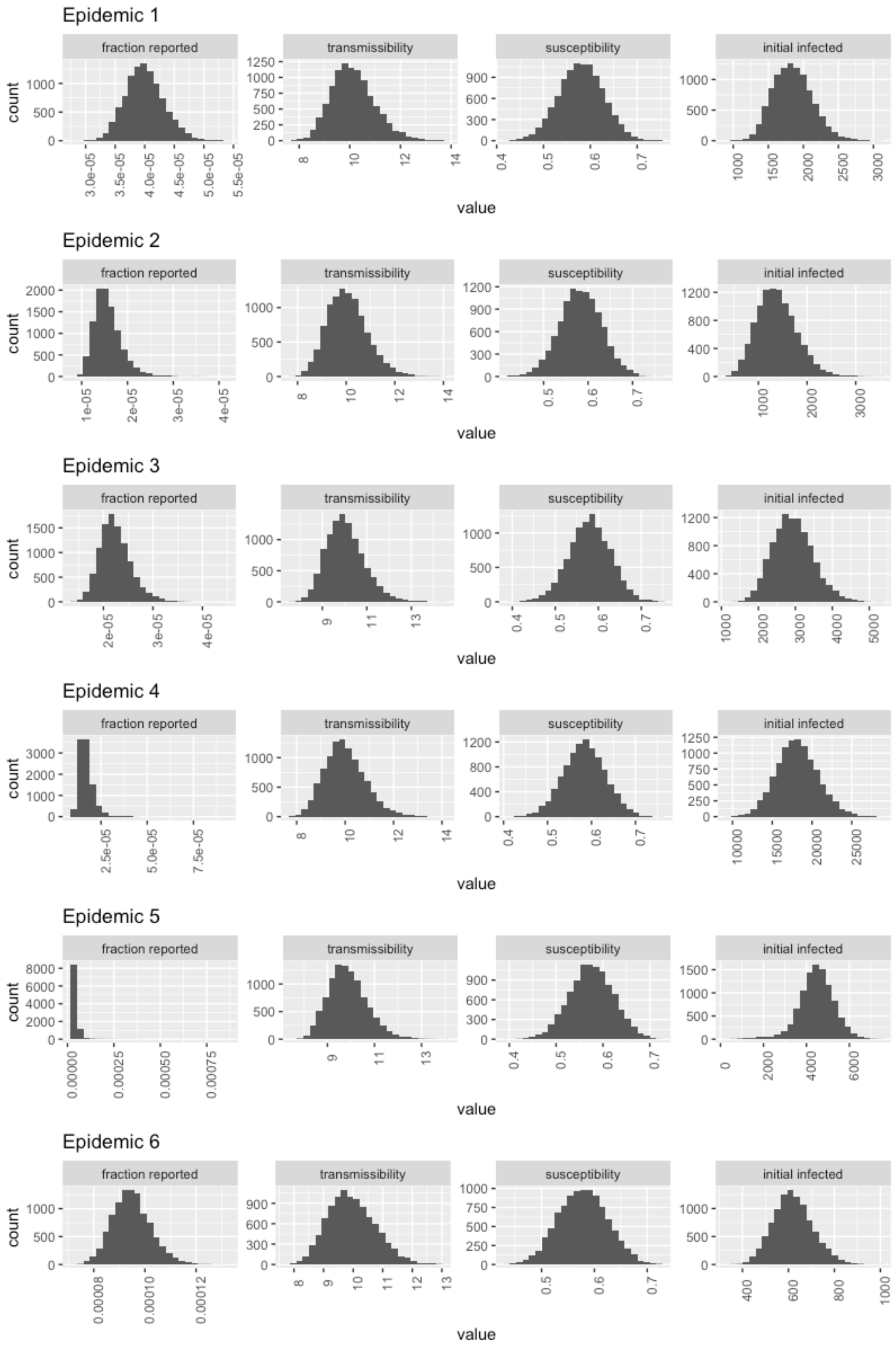

Supplementary Figure 3: Posterior distributions of fitted parameters for each epidemic-period.

B.3.4 Simulations combining epidemic and non-epidemic periods.

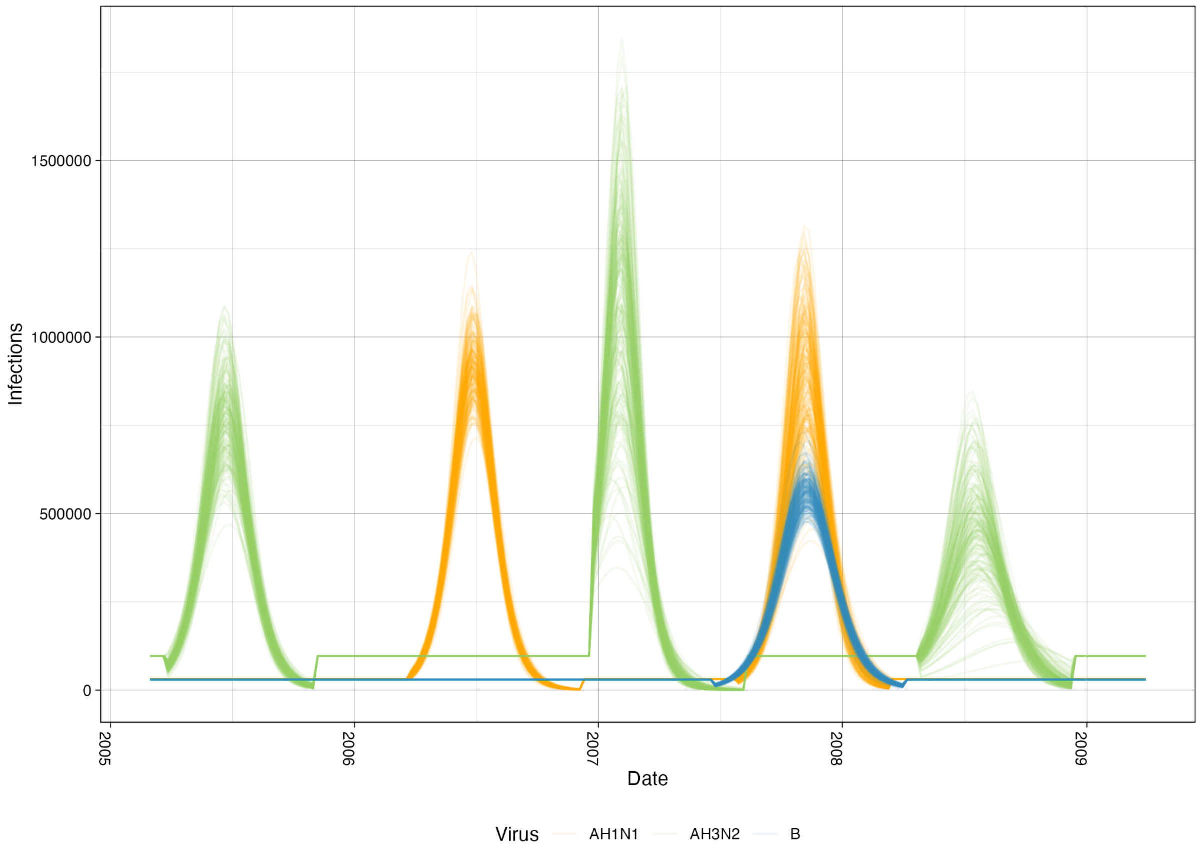

Supplementary Figure 4: Simulated number of influenza infections combining epidemic and non-epidemic periods for different influenza subtypes in the absence of vaccination.

B.4 Risk of death given symptomatic infection

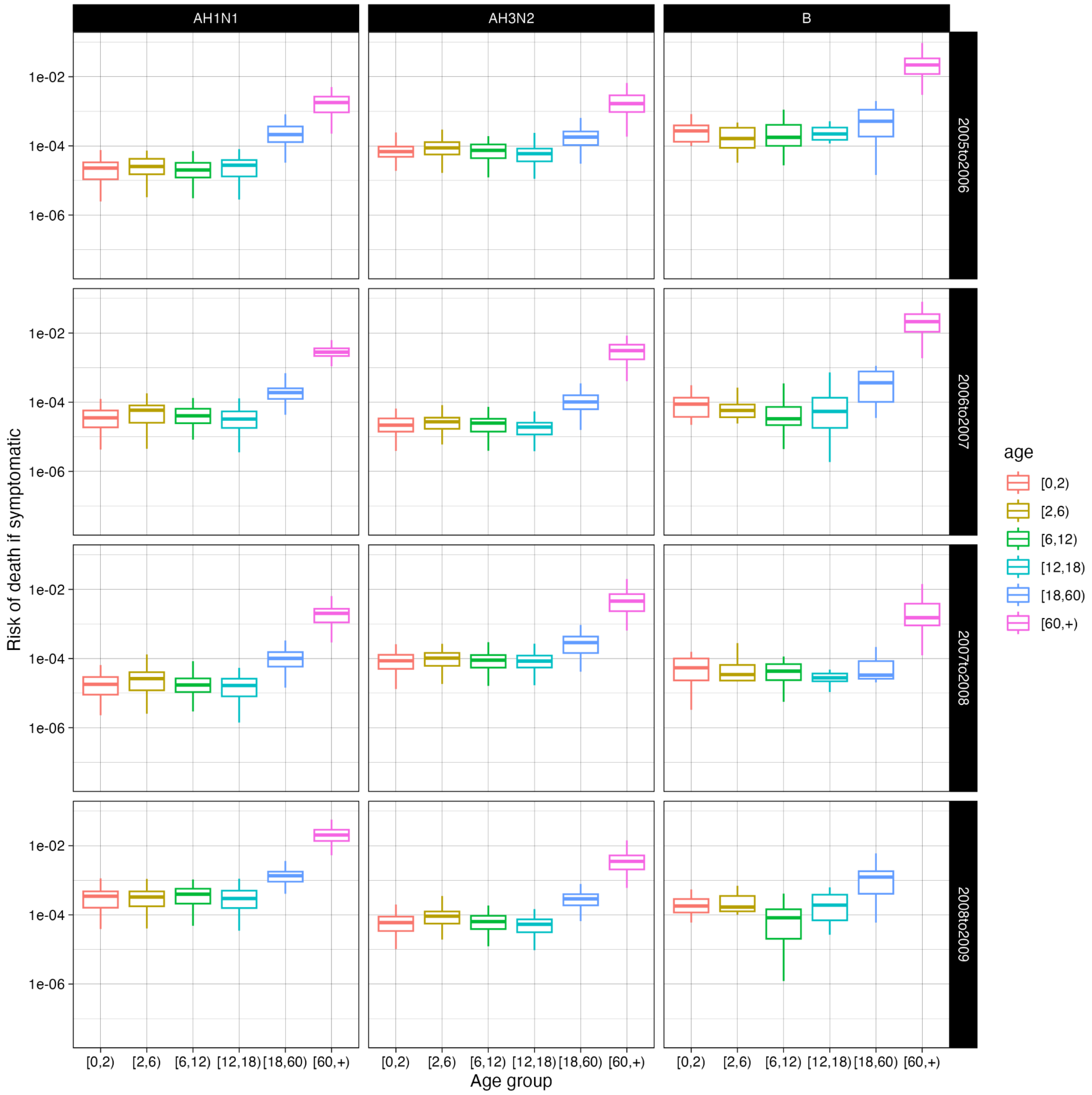

Supplementary Figure 5: Calculated age- and subtype-specific risk of death amongst symptomatic cases calculated by combining the modelled number of symptomatic with data on the number of deaths from Meeyai et al.

C. Supplementary results

C.1 Vaccine doses given under different scenarios

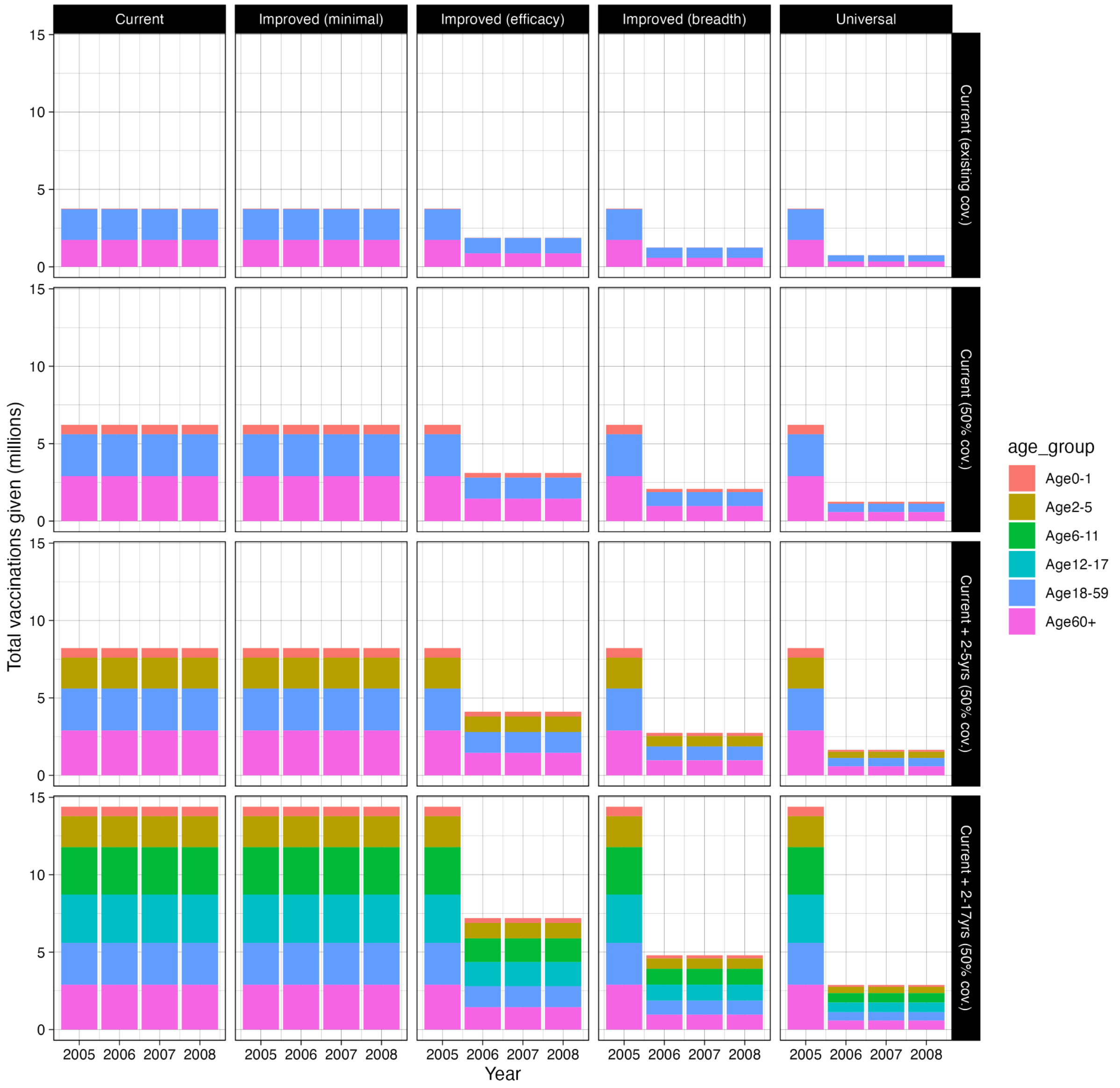

Supplementary Figure 6: Number of vaccine doses given annually by vaccine scenario and coverage scenario (see table 1).

C.2 Impact of different vaccine and coverage scenarios

Supplementary Table 4: Cumulative number of infections, outpatient cases, inpatient cases, deaths and DALYs for different NGIVs and coverage scenarios.

Vaccine scenario	Outcome	No vaccine	Current programme (existing coverage)	Current programme (50% coverage)	Current programme + 2-5 year olds (50% coverage)	Current programme + 2-17 year olds (50% coverage)
**No vaccine**	infections	7.43e+07 (6.72e+07, 8e+07)				
**Current**	infections		6.12e+07 (5.46e+07, 6.67e+07)	5.51e+07 (4.89e+07, 6.03e+07)	5.12e+07 (4.53e+07, 5.62e+07)	3.43e+07 (3.02e+07, 3.79e+07)
**Improved (minimal)**	infections		5.69e+07 (5.05e+07, 6.22e+07)	4.94e+07 (4.36e+07, 5.44e+07)	4.46e+07 (3.94e+07, 4.92e+07)	2.95e+07 (2.63e+07, 3.26e+07)
**Improved (efficacy)**	infections		5.09e+07 (4.49e+07, 5.6e+07)	4.15e+07 (3.64e+07, 4.6e+07)	3.6e+07 (3.17e+07, 3.97e+07)	2.35e+07 (2.22e+07, 2.49e+07)
**Improved (breadth)**	infections		5.36e+07 (4.73e+07, 5.87e+07)	4.48e+07 (3.94e+07, 4.96e+07)	3.88e+07 (3.43e+07, 4.31e+07)	2.35e+07 (2.23e+07, 2.48e+07)
**Universal**	infections		4.85e+07 (4.27e+07, 5.34e+07)	3.88e+07 (3.42e+07, 4.31e+07)	3.28e+07 (2.93e+07, 3.62e+07)	2.12e+07 (2.05e+07, 2.2e+07)
**No vaccine**	Deaths	1.99e+04 (1.31e+04, 2.85e+04)				
**Current**	Deaths		1.64e+04 (1.05e+04, 2.38e+04)	1.5e+04 (9.47e+03, 2.2e+04)	1.45e+04 (9.12e+03, 2.13e+04)	1.17e+04 (6.84e+03, 1.78e+04)
**Improved (minimal)**	Deaths		1.53e+04 (9.8e+03, 2.24e+04)	1.36e+04 (8.55e+03, 2.01e+04)	1.29e+04 (7.93e+03, 1.91e+04)	1.02e+04 (5.91e+03, 1.55e+04)
**Improved (efficacy)**	Deaths		1.37e+04 (8.9e+03, 2.02e+04)	1.15e+04 (7.17e+03, 1.7e+04)	1.06e+04 (6.48e+03, 1.58e+04)	8.41e+03 (4.86e+03, 1.3e+04)
**Improved (breadth)**	Deaths		1.44e+04 (9.46e+03, 2.1e+04)	1.22e+04 (7.9e+03, 1.79e+04)	1.13e+04 (7.08e+03, 1.66e+04)	8.49e+03 (4.9e+03, 1.3e+04)
**Universal**	Deaths		1.31e+04 (8.55e+03, 1.91e+04)	1.05e+04 (6.72e+03, 1.56e+04)	9.59e+03 (5.92e+03, 1.42e+04)	7.61e+03 (4.46e+03, 1.18e+04)
**No vaccine**	Inpatient cases	7.82e+03 (5.99e+03, 1e+04)				
**Current**	Inpatient cases		6.47e+03 (4.94e+03, 8.33e+03)	5.82e+03 (4.44e+03, 7.49e+03)	5.36e+03 (4.07e+03, 6.88e+03)	3.5e+03 (2.69e+03, 4.52e+03)
**Improved (minimal)**	Inpatient cases		6.03e+03 (4.6e+03, 7.75e+03)	5.23e+03 (3.96e+03, 6.73e+03)	4.65e+03 (3.53e+03, 5.99e+03)	2.97e+03 (2.29e+03, 3.82e+03)

**Improved (efficacy)**	Inpatient cases		5.42e+03 (4.11e+03, 6.98e+03)	4.4e+03 (3.34e+03, 5.7e+03)	3.71e+03 (2.81e+03, 4.81e+03)	2.32e+03 (1.83e+03, 2.93e+03)
**Improved (breadth)**	Inpatient cases		5.7e+03 (4.33e+03, 7.36e+03)	4.78e+03 (3.6e+03, 6.16e+03)	4.05e+03 (3.08e+03, 5.24e+03)	2.33e+03 (1.83e+03, 2.94e+03)
**Universal**	Inpatient cases		5.18e+03 (3.92e+03, 6.7e+03)	4.14e+03 (3.11e+03, 5.37e+03)	3.38e+03 (2.58e+03, 4.36e+03)	2.08e+03 (1.64e+03, 2.6e+03)
**No vaccine**	Outpatient cases	3.51e+04 (2.13e+04, 5.34e+04)				
**Current**	Outpatient cases		2.89e+04 (1.77e+04, 4.38e+04)	2.61e+04 (1.59e+04, 3.95e+04)	2.4e+04 (1.46e+04, 3.68e+04)	1.61e+04 (9.61e+03, 2.47e+04)
**Improved (minimal)**	Outpatient cases		2.69e+04 (1.65e+04, 4.08e+04)	2.34e+04 (1.43e+04, 3.56e+04)	2.09e+04 (1.27e+04, 3.17e+04)	1.38e+04 (8.14e+03, 2.12e+04)
**Improved (efficacy)**	Outpatient cases		2.41e+04 (1.48e+04, 3.68e+04)	1.97e+04 (1.2e+04, 3.01e+04)	1.69e+04 (1.01e+04, 2.57e+04)	1.09e+04 (6.46e+03, 1.69e+04)
**Improved (breadth)**	OOutpatient cases		2.54e+04 (1.56e+04, 3.86e+04)	2.13e+04 (1.31e+04, 3.27e+04)	1.83e+04 (1.1e+04, 2.8e+04)	1.09e+04 (6.49e+03, 1.69e+04)
**Universal**	Outpatient cases		2.3e+04 (1.41e+04, 3.52e+04)	1.84e+04 (1.13e+04, 2.83e+04)	1.54e+04 (9.23e+03, 2.35e+04)	9.78e+03 (5.83e+03, 1.53e+04)
**No vaccine**	DALYs (discounted)	4.64e+05 (3.12e+05, 6.69e+05)				
**Current**	DALYs (discounted)		3.85e+05 (2.57e+05, 5.59e+05)	3.5e+05 (2.34e+05, 5.08e+05)	3.31e+05 (2.23e+05, 4.79e+05)	2.43e+05 (1.63e+05, 3.48e+05)
**Improved (minimal)**	DALYs (discounted)		3.61e+05 (2.41e+05, 5.24e+05)	3.17e+05 (2.13e+05, 4.6e+05)	2.93e+05 (1.97e+05, 4.19e+05)	2.12e+05 (1.43e+05, 3.01e+05)
**Improved (efficacy)**	DALYs (discounted)		3.26e+05 (2.19e+05, 4.71e+05)	2.71e+05 (1.82e+05, 3.88e+05)	2.42e+05 (1.62e+05, 3.44e+05)	1.73e+05 (1.16e+05, 2.46e+05)
**Improved (breadth)**	DALYs (discounted)		3.42e+05 (2.27e+05, 4.97e+05)	2.9e+05 (1.97e+05, 4.18e+05)	2.59e+05 (1.75e+05, 3.73e+05)	1.74e+05 (1.17e+05, 2.47e+05)
**Universal**	DALYs (discounted)		3.11e+05 (2.09e+05, 4.51e+05)	2.53e+05 (1.71e+05, 3.64e+05)	2.2e+05 (1.49e+05, 3.18e+05)	1.58e+05 (1.05e+05, 2.27e+05)
**No vaccine**	DALYs	4.86e+05 (3.27e+05, 7.01e+05)				
**Current**	DALYs		4.03e+05 (2.7e+05, 5.85e+05)	3.67e+05 (2.46e+05, 5.32e+05)	3.46e+05 (2.33e+05, 5e+05)	2.53e+05 (1.72e+05, 3.62e+05)
**Improved (minimal)**	DALYs		3.77e+05 (2.52e+05, 5.47e+05)	3.32e+05 (2.23e+05, 4.81e+05)	3.06e+05 (2.06e+05, 4.38e+05)	2.21e+05 (1.49e+05, 3.12e+05)

**Improved (efficacy)**	DALYs		3.41e+05 (2.28e+05, 4.94e+05)	2.83e+05 (1.9e+05, 4.07e+05)	2.53e+05 (1.69e+05, 3.59e+05)	1.81e+05 (1.21e+05, 2.58e+05)
**Improved (breadth)**	DALYs		3.57e+05 (2.4e+05, 5.2e+05)	3.03e+05 (2.06e+05, 4.38e+05)	2.7e+05 (1.84e+05, 3.87e+05)	1.81e+05 (1.22e+05, 2.57e+05)
**Universal**	DALYs		3.25e+05 (2.18e+05, 4.72e+05)	2.64e+05 (1.79e+05, 3.8e+05)	2.3e+05 (1.56e+05, 3.3e+05)	1.65e+05 (1.1e+05, 2.37e+05)
**No vaccine**	DALYs averted	NA				
**Current**	DALYs (incremental)		0 (0, 0)	0 (0, 0)	0 (0, 0)	0 (0, 0)
**Improved (minimal)**	DALYs (incremental)		-2.47e+04 (-3.86e+04, -1.59e+04)	-3.22e+04 (-5.01e+04, -2.06e+04)	-3.74e+04 (-5.77e+04, -2.38e+04)	-3.06e+04 (-4.68e+04, -1.93e+04)
**Improved (efficacy)**	DALYs (incremental)		-5.85e+04 (-9.06e+04, -3.76e+04)	-7.87e+04 (-1.19e+05, -5.11e+04)	-8.95e+04 (-1.33e+05, -5.82e+04)	-6.91e+04 (-1.05e+05, -4.4e+04)
**Improved (breadth)**	DALYs (incremental)		-4.27e+04 (-6.58e+04, -2.71e+04)	-5.9e+04 (-8.95e+04, -3.79e+04)	-7.09e+04 (-1.08e+05, -4.57e+04)	-6.85e+04 (-1.05e+05, -4.32e+04)
**Universal**	DALYs (incremental)		-7.23e+04 (-1.11e+05, -4.64e+04)	-9.64e+04 (-1.44e+05, -6.26e+04)	-1.1e+05 (-1.62e+05, -7.21e+04)	-8.43e+04 (-1.28e+05, -5.37e+04)
**No vaccine**	Vaccine costs	NA				
**Current**	Vaccine costs		1.19e+08 (1.02e+08, 1.4e+08)	1.96e+08 (1.69e+08, 2.31e+08)	2.6e+08 (2.24e+08, 3.05e+08)	4.04e+08 (3.4e+08, 5.57e+08)
**Improved (minimal)**	Vaccine costs		1.19e+08 (1.02e+08, 1.4e+08)	1.96e+08 (1.69e+08, 2.31e+08)	2.6e+08 (2.24e+08, 3.05e+08)	4.04e+08 (3.4e+08, 5.57e+08)
**Improved (efficacy)**	Vaccine costs		7.43e+07 (6.4e+07, 8.74e+07)	1.23e+08 (1.06e+08, 1.44e+08)	1.62e+08 (1.4e+08, 1.91e+08)	2.53e+08 (2.13e+08, 3.48e+08)
**Improved (breadth)**	Vaccine costs		5.94e+07 (5.12e+07, 6.99e+07)	9.82e+07 (8.46e+07, 1.16e+08)	1.3e+08 (1.12e+08, 1.53e+08)	2.02e+08 (1.7e+08, 2.78e+08)
**Universal**	Vaccine costs		4.75e+07 (4.1e+07, 5.59e+07)	7.85e+07 (6.77e+07, 9.24e+07)	1.04e+08 (8.95e+07, 1.22e+08)	1.62e+08 (1.36e+08, 2.23e+08)
**No vaccine**	Total costs	2.02e+07 (1.62e+07, 2.45e+07)				
**Current**	Total costs		1.35e+08 (1.19e+08, 1.57e+08)	2.11e+08 (1.83e+08, 2.46e+08)	2.73e+08 (2.37e+08, 3.19e+08)	4.13e+08 (3.48e+08, 5.66e+08)
**Improved (minimal)**	Total costs		1.34e+08 (1.17e+08, 1.56e+08)	2.1e+08 (1.82e+08, 2.45e+08)	2.72e+08 (2.35e+08, 3.18e+08)	4.12e+08 (3.47e+08, 5.64e+08)

**Improved (efficacy)**	Total costs		8.83e+07 (7.77e+07, 1.02e+08)	1.34e+08 (1.17e+08, 1.56e+08)	1.72e+08 (1.49e+08, 2.01e+08)	2.59e+08 (2.18e+08, 3.54e+08)
**Improved (breadth)**	Total costs		7.42e+07 (6.55e+07, 8.51e+07)	1.1e+08 (9.65e+07, 1.28e+08)	1.4e+08 (1.22e+08, 1.63e+08)	2.08e+08 (1.75e+08, 2.84e+08)
**Universal**	Total costs		6.1e+07 (5.38e+07, 6.99e+07)	8.93e+07 (7.81e+07, 1.03e+08)	1.13e+08 (9.78e+07, 1.31e+08)	1.67e+08 (1.41e+08, 2.28e+08)

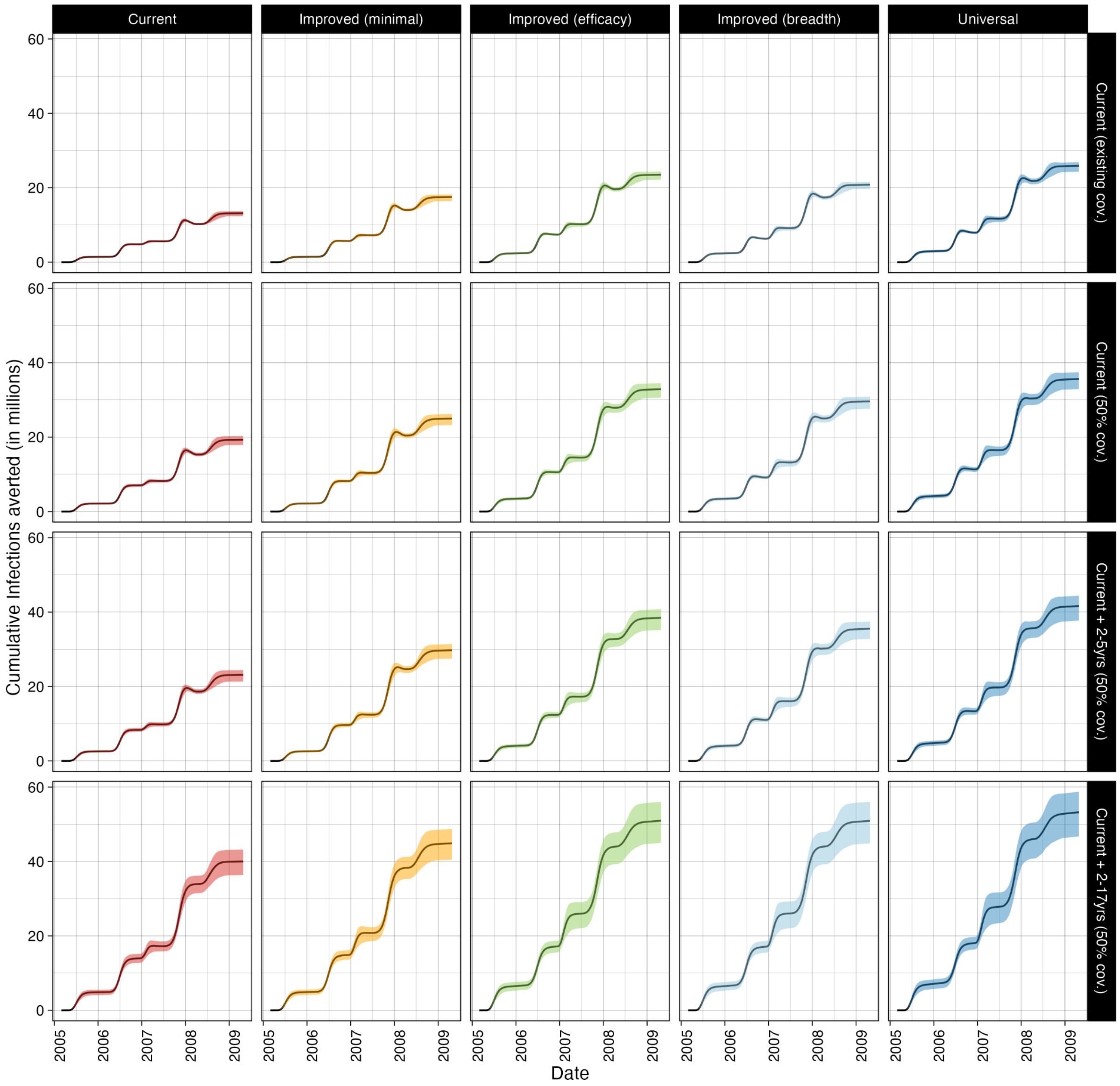

Supplementary Figure 7: Cumulative number of infections averted by different NGIVs under different vaccine scenarios and coverage scenarios.

C.3 Cost-Effectiveness

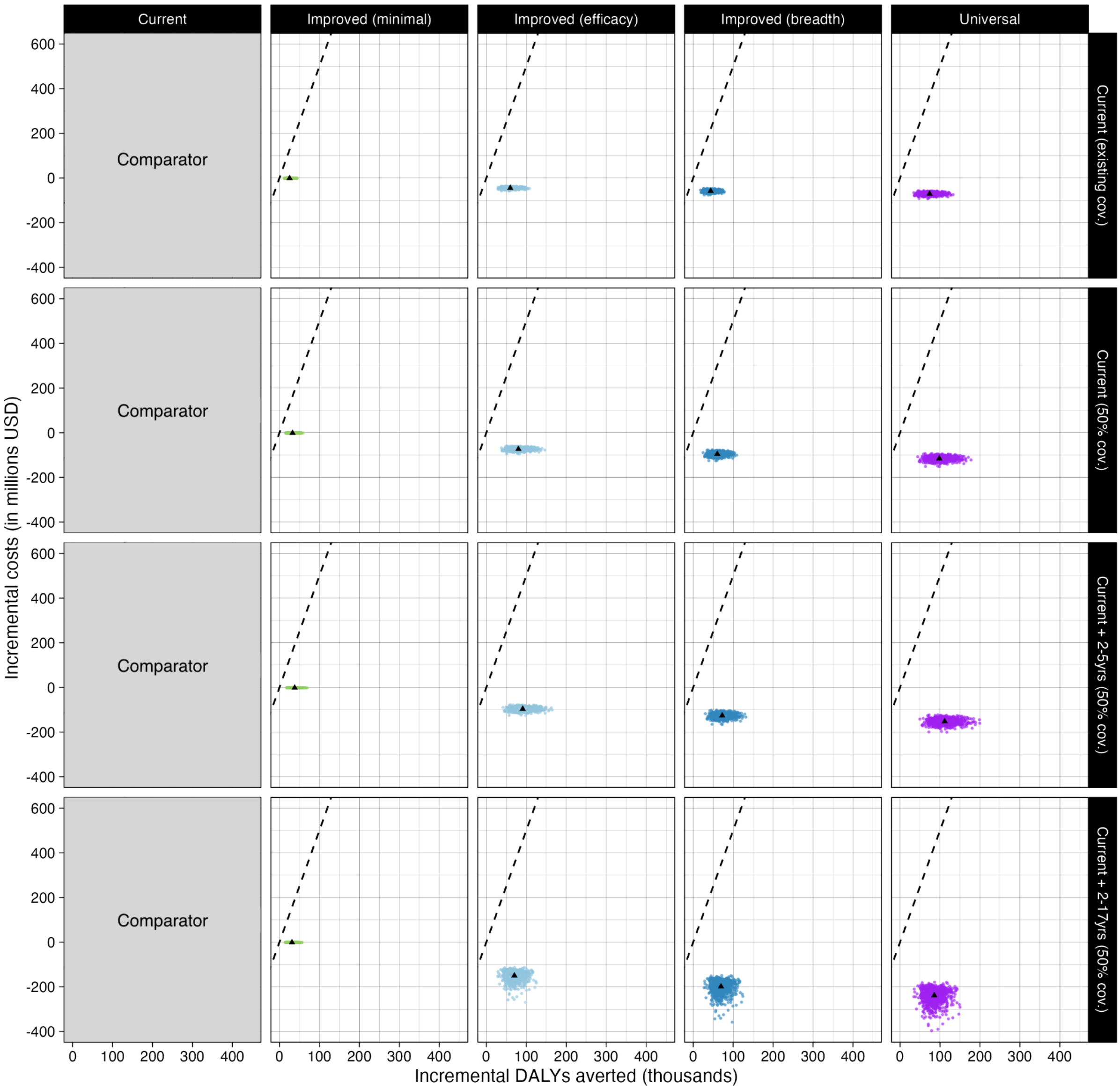

Supplementary Figure 8: Cost-effectiveness plane showing incremental costs and incremental DALYs averted for different vaccine types when comparing NGIVs under different coverage scenarios with the *equivalent coverage of current vaccines* (comparator (ii) in table 1). Dashed lines show the guideline cost-effectiveness threshold of 160,000 Baht per DALY averted.

C.4 Net Monetary Benefit

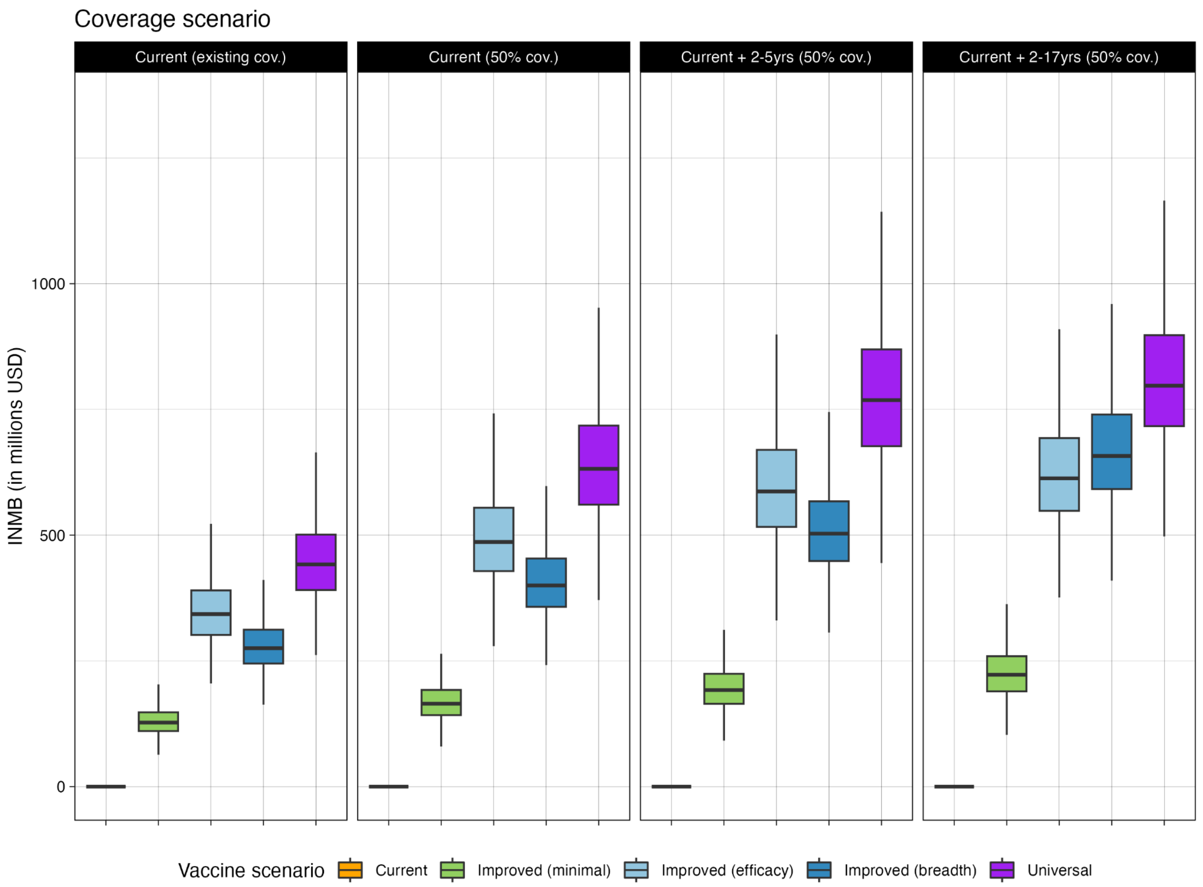

Supplementary Figure 9: Incremental Net Monetary Benefit of different NGIV vaccine types and coverage strategies with 3% DALY discounting when comparing NGIVs under different coverage scenarios with the *equivalent coverage of current vaccines* (comparator (ii) in table 1).

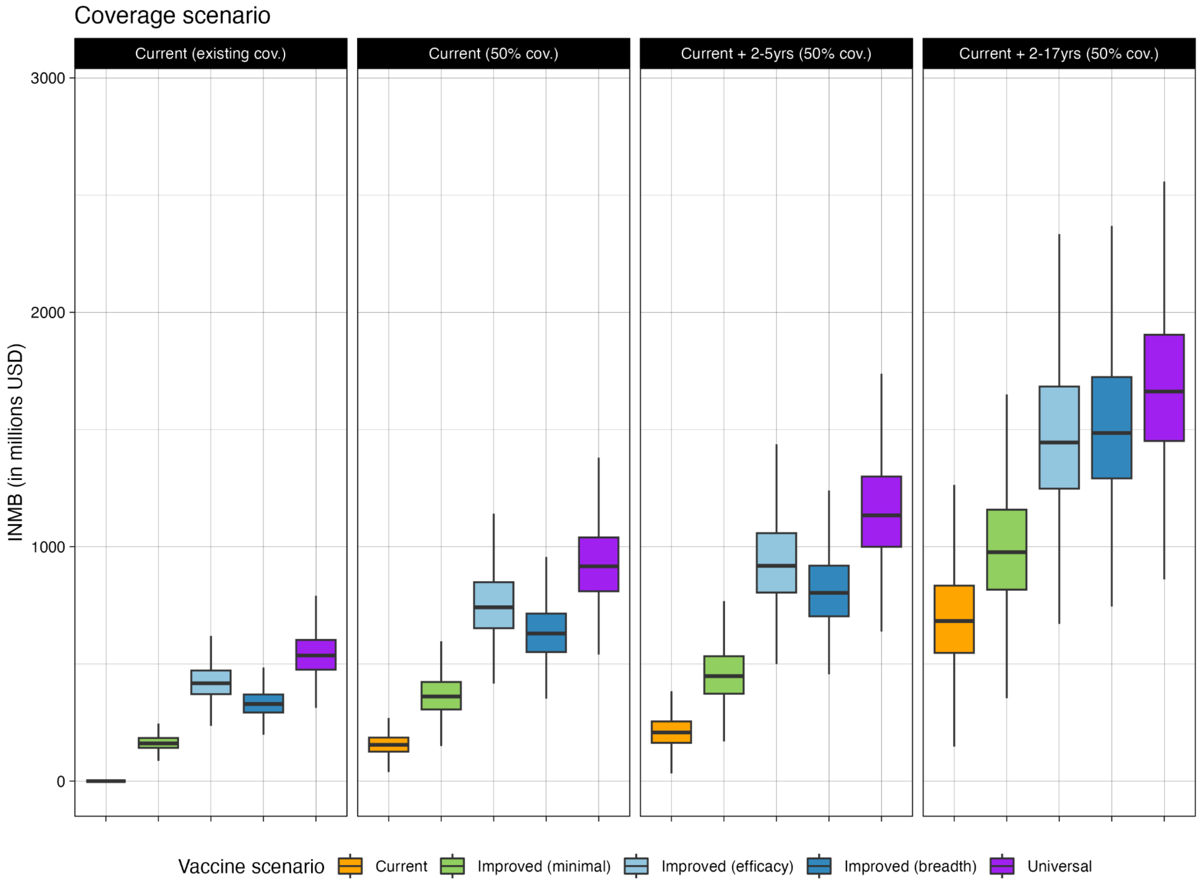

Supplementary Figure 10: Incremental Net Monetary Benefit of different NGIV vaccine types under different coverage scenarios compared to *existing coverage of current vaccines* using 0% annual discounting of future DALYs (comparator (i) in table 1).

C.5 Threshold Vaccine Prices

Supplementary Table 5: Sensitivity analysis using 0% discounting of future DALYs showing threshold prices in United States Dollars below which different types of NGIV and coverage scenarios become cost-effective using the recommended cost-effectiveness threshold in Thailand of 160,000 per DALY averted. Threshold prices are presented based on two comparators: (i) existing coverage of current vaccines, and (ii) coverage of current vaccines that corresponds to the NGIV scenario.

Comparator	NGIV scenario	Coverage Scenario			
		S1 - Current age-targeting (existing coverage)	S2 - Current age-targeting (50% coverage)	S3 - Current age-targeting + 2-5 year olds (50% coverage)	S4 - Current age-targeting + 2-17 year olds (50% coverage)
(i) Current vaccines and current coverage (scenarios S1)	Current	*Comparator*	7.77 (4.44, 12.1)	8.33 (4.47, 13.5)	14.8 (8.11, 23.2)
	Improved (minimal)	11.2 (7.32, 16.6)	16.4 (10.2, 25)	16 (9.65, 25.1)	20.1 (12.1, 30.9)
	Improved (efficacy)	44.1 (29.8, 64.1)	49.5 (33.1, 73)	47.2 (31.3, 70.3)	43.4 (28.9, 63.8)
	Improved (breadth)	41.9 (29.1, 60.1)	51.5 (34, 75.4)	50.6 (33.7, 76.1)	54.7 (36.8, 80.1)
	Universal	86.4 (59.1, 124)	92.6 (63.3, 135)	87.7 (60.1, 128)	74.9 (51.1, 109)
(ii) Current vaccines with the same coverage scenario (i.e. comparing within the same column)	Current	*Comparator*	*Comparator*	*Comparator*	*Comparator*
	Improved (minimal)	11.2 (7.32, 16.6)	8.64 (5.57, 13.1)	7.59 (4.88, 11.6)	5.36 (3.56, 8.08)
	Improved (efficacy)	44.1 (29.8, 64.1)	37.4 (25.6, 54.8)	34.2 (24, 48.6)	20.1 (14.4, 28.1)
	Improved (breadth)	41.9 (29.1, 60.1)	36.2 (25.2, 51.4)	34.4 (24.5, 48.6)	25.7 (18.3, 35.7)
	Universal	86.4 (59.1, 124)	73.9 (51.9, 105)	67.5 (48.5, 95.2)	38.8 (27.8, 53.7)
